# Thiazole: A Versatile Standalone Moiety Contributing to the Development of Various Drugs and Biologically Active Agents

**DOI:** 10.3390/molecules27133994

**Published:** 2022-06-21

**Authors:** Mohammed F. Arshad, Aftab Alam, Abdullah Ayed Alshammari, Mohammed Bader Alhazza, Ibrahim Mohammed Alzimam, Md Anish Alam, Gulam Mustafa, Md Salahuddin Ansari, Abdulelah M. Alotaibi, Abdullah A. Alotaibi, Suresh Kumar, Syed Mohammed Basheeruddin Asdaq, Mohd. Imran, Pran Kishore Deb, Katharigatta N. Venugopala, Shahamah Jomah

**Affiliations:** 1 Department of Research and Scientific Communications, Isthmus Research and Publishing House, U-13, Near Badi Masjid, Pulpehlad Pur, New Delhi 110044, India; anish@isthmuspub.com; 2 Department of Pharmacognosy, College of Pharmacy, Prince Sattam Bin Abdulaziz University, Al-Kharj 11942, Saudi Arabia; a.alam@psau.edu.sa; 3 Faculty of Pharmacy, Northern Border University, Rafha 91911, Saudi Arabia; abdullahayed1419@yahoo.com (A.A.A.); a1hazza774@gmail.com (M.B.A.); ibrah11eem@gmail.com (I.M.A.); 4 Department of Pharmaceutical Sciences, College of Pharmacy (Al-Dawadmi Campus), Shaqra University, Riyadh 11961, Saudi Arabia; gulampharma@gmail.com; 5 Department of Pharmacy Practice, College of Pharmacy (Al-Dawadmi Campus), Shaqra University, Riyadh 11961, Saudi Arabia; msalahuddin@su.edu.sa; 6 Internee, College of Pharmacy (Al-Dawadmi Campus), Shaqra University, Riyadh 11961, Saudi Arabia; abdullelah.i97@gmail.com (A.M.A.); abdullahabdulrhman.o@gmail.com (A.A.A.); 7 Drug Regulatory Affair, Department, Pharma Beistand, New Delhi 110017, India; sureshkr.2006@gmail.com; 8 Department of Pharmacy Practice, College of Pharmacy, AlMaarefa University, Dariyah 13713, Saudi Arabia; 9 Department of Pharmaceutical Chemistry, Faculty of Pharmacy, Northern Border University, Rafha 91911, Saudi Arabia; 10 Department of Pharmaceutical Sciences, Faculty of Pharmacy, Philadelphia University, Amman 19392, Jordan; prankishore1@gmail.com; 11 Department of Pharmaceutical Sciences, College of Clinical Pharmacy, King Faisal University, Al-Ahsa 31982, Saudi Arabia; kvenugopala@kfu.edu.sa; 12 Department of Biotechnology and Food Science, Faculty of Applied Sciences, Durban University of Technology, Durban 4001, South Africa; 13 Pharmacy Department, Dr. Sulaiman Al-Habib Medical Group, Riyadh 11372, Saudi Arabia; shahama.joma@gmail.com

**Keywords:** thiazole, sulfur, nitrogen, oxidation reaction, aromaticity, donor-acceptor reaction

## Abstract

For many decades, the thiazole moiety has been an important heterocycle in the world of chemistry. The thiazole ring consists of sulfur and nitrogen in such a fashion that the pi (π) electrons are free to move from one bond to other bonds rendering aromatic ring properties. On account of its aromaticity, the ring has many reactive positions where donor–acceptor, nucleophilic, oxidation reactions, etc., may take place. Molecules containing a thiazole ring, when entering physiological systems, behave unpredictably and reset the system differently. These molecules may activate/stop the biochemical pathways and enzymes or stimulate/block the receptors in the biological systems. Therefore, medicinal chemists have been focusing their efforts on thiazole-bearing compounds in order to develop novel therapeutic agents for a variety of pathological conditions. This review attempts to inform the readers on three major classes of thiazole-bearing molecules: Thiazoles as treatment drugs, thiazoles in clinical trials, and thiazoles in preclinical and developmental stages. A compilation of preclinical and developmental thiazole-bearing molecules is presented, focusing on their brief synthetic description and preclinical studies relating to structure-based activity analysis. The authors expect that the current review may succeed in drawing the attention of medicinal chemists to finding new leads, which may later be translated into new drugs.

## 1. Introduction

In the list of five-membered heteroaryl ring systems, thiazole is a unique ring that carries nitrogen and sulfur atoms, which makes it a versatile entity in actions and reactions. Though free thiazole cannot be spotted in nature, the ring of thiazole is detected in several natural compounds, such as peptide alkaloids, metabolites, and cyclopeptides [1]. The lonely pair of electrons in the sulfur atom of the thiazole ring is dislocated, which meets the Huckel rule condition for a minimum of six pi (π) electrons [2]. Thiazole undergoes various reactions such as donor–acceptor [3], intramolecular nucleophilic substitution [4], photochemical reaction [5,6], arylation [7], cycloaddition [8], oxidation [9], transformation [10], dimerization [11], etc. Some old but important methods for the production of thiazole rings include Hantzsch thiazole synthesis [12,13], Cook–Heilbron synthesis [14], Herz synthesis [15], modified Hantzsch synthesis [16], etc. Recently, thiazole derivatives have been synthesized by Venugopala (2017) [17]. Thiazole ring bears an acidic proton at C-2, due to which thiazole ring becomes highly reactive and has evolved as a significant synthon for the production of a wide range of new chemical compounds. Derivatives of thiazole have always piqued the interest of synthetic and biological chemists due to their various chemical, physical, and pharmacological properties. A variety of new compounds with a diverse spectrum of therapeutic potentials such as antioxidant, anti-tubercular, antibacterial, antifungal, diuretic, anti-cancer, and anti-inflammatory effects were produced by modifying the thiazole ring at various locations [18]. Thiazole, as a single nucleus or fused ring, is a key component of natural penicillin-like drugs known as antibiotics.

The thiazole moiety has received much attention in recent decades, with multiple review articles stressing the significance of the thiazole nucleus in the design and optimization of newer bioactive drug candidates [19,20,21,22,23,24,25]. All these research articles highlighted the preliminary aspect of thiazole moiety, and none of them presented a chronological significance of thiazole moiety. In the current review article, the authors are trying to inform the readers of some of the new dimensions of the thiazole platform, for example, thiazole-ring-bearing drugs, which are clinically used in various diseases, thiazole-containing drug candidates that are in clinical trial phases or in the preclinical developmental state, etc. The authors expect that the current review article may succeed in drawing the attention of medicinal chemists to finding some new leads, which will be optimized to generate a new drug. 

## 2. Thiazole-Derived Treatment Drugs

**Figure 1:**
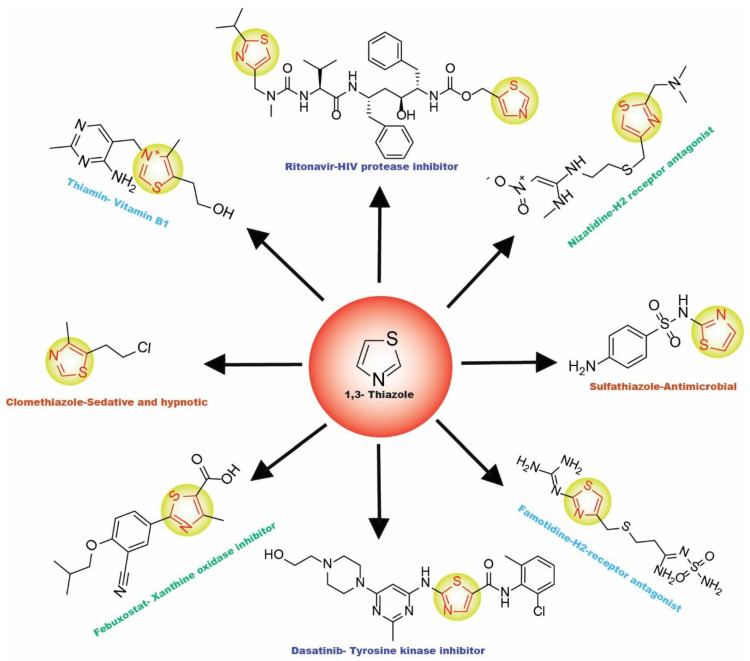
Some of the active pharmaceutical ingredients bearing thiazole ring.

**Figure 2:**
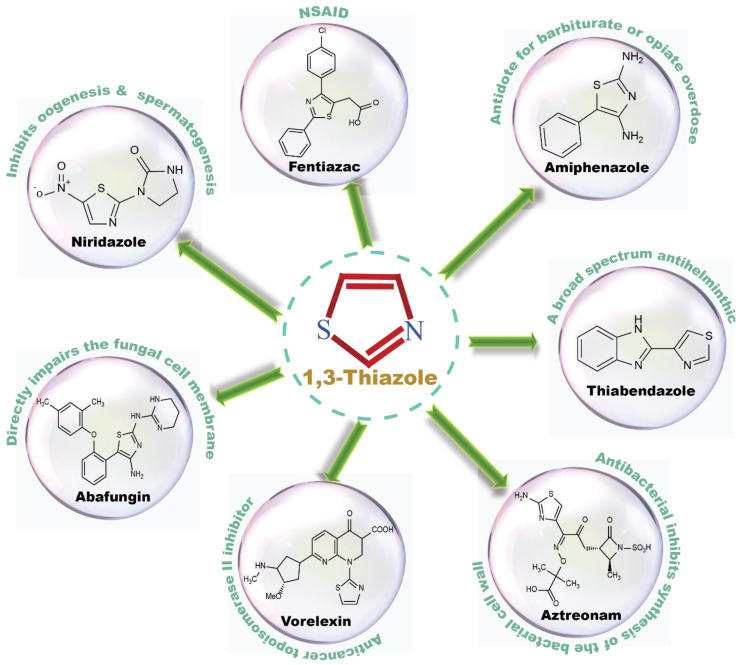
Some of the active pharmaceutical ingredients bearing thiazole ring.

The thiazole ring can be detected in a wide collection of natural or synthetic molecules with varying degree of biological activity. The thiazole-containing vitamin B1 (thiamine), for example, aids in the regular working of the neurological system by facilitating the formation of acetylcholine [26]. Some thiazole analogues have previously been employed as effective central nervous system (CNS) medications. Pramipexole, which contains 2-amino-thiazole moiety linked to a cyclohexane ring structurally similar to dopamine’s catechol ring, exhibited dopamine D2 agonist activity and was used in the treatment of Parkinson’s disease [27]. Riluzole, an aminothiazole-based drug, has been licensed to manage Lou Gehrig’s disease [28]. Ritonavir is a protease suppressor antiretroviral medication used to curb HIV contagion [29]. Nizatidine is a histamine-2 (H_2_R) receptor suppressor that reduces the formation of stomach acid and is often used to control peptic ulcers and gastroesophageal reflux disease [30]. Sulfathiazole is an organosulfur sulfa drug prescribed for a short duration to treat microbial infections [31]. Famotidine lessens the formation of stomach acid by blocking the histamine-2 (H_2_R) receptor [32]. Dasatinib is a tyrosine kinase suppressor that inhibits both Abelson murine leukemia and sarcoma Abl and Src. It has been found to have immunomodulatory properties. The medication has been approved to manage chronic myeloid leukemia [33]. A nonpurine-specific suppressor of the enzyme xanthine oxidase, febuxostat, is used to manage gout-associated hyperuricemia [34]. Clomethiazole is a drug that is prescribed to address sleep problems [35]. Niridazole is used as schistosomicide and also is prescribed for the treatment of periodontitis (inflammatory disease) [36,37]. Joint and muscular discomforts are managed with a newly introduced non-steroidal drug, fentiazac [38]. In the cure of barbiturate or opiate poisoning, amiphenazole is employed [39]. The antifungal medication abafungin is mostly used topically to suppress skin infections caused by various fungi [40]. Voreloxin binds to DNA and interacts with topoisomerase II, resulting in DNA double-strand cracks, a G2 stop, and ultimately, cell death. Voreloxin is presently in use for the management of various neoplasms [41]. Aztreonam, also known as azactam, is a penicillin antibiotic administered to control the conditions due to Gram-negative bacterial contamination [42]. Thiabendazole inhibits the helminth-specific mitochondrial enzyme, stopping the tricarboxylic acid cycle (TCA cycle) and adenosine triphosphate (ATP) production leading to helminth’s death [43] (Figure 1 and Figure 2).

## 3. Thiazole Bearing Drug Candidates under Intensive Clinical Investigations

As mentioned in an earlier section, many more drug-bearing thiazole rings are nowadays used in the management of various diseases, and medicinal chemists across the globe are still hopeful in finding some of the best medications from this class. Research has been ongoing, and a host of potential drug molecules have been customized for clinical use. Some of them have completed clinical trials and are in the final stage of receiving approval from regulatory bodies, and some are in the process of entering clinical trials. As shown in Table 1, we have outlined several therapeutic candidates who have the potential to become treatments for several ailments in the coming days.

## 4. Thiazole-Bearing Compounds in Pre-Clinical Investigations

### 4.1. Anticonvulsant Activity

**Figure 3:**
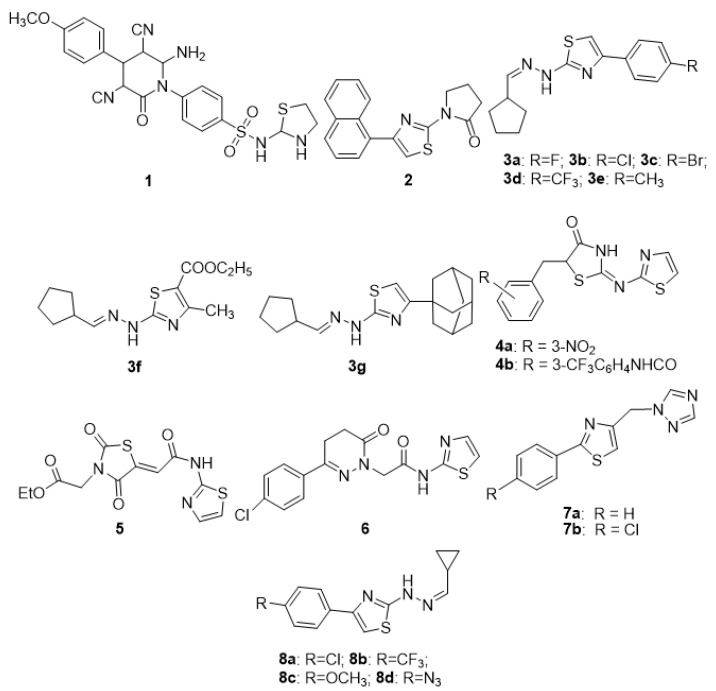
Recently planned and produced thiazoles as potential anticonvulsant agents.

Epilepsy is a set of neurological disorders. In all types of epilepsies, unprovoked, excessive, sudden, self-regulated neuronal firing occurs, resulting in a seizure. The finely structured pattern of the brain’s integrative activity is lost due to excessive neuronal discharge. The condition is treated with palliative care, and resistance to the medication develops with time. Furthermore, current medicines have a slew of side effects, forcing the development of a new class of pharmaceuticals to address these concerns. Thiazole is a well-famed platform for the development of various bioactive compounds of diverse classes. Current research trends show that numerous newly developed thiazole agents with high lipophilicity are capable of stopping seizures to the same extent as treatment with medicine [57,58]. Figure 3 shows some of the recently synthesized potent anticonvulsant agents.

Farag et al. (2012) synthesized new heterocyclic compounds with the starting compound 4-amino-N-(thiazol-2-yl)benzene sulfonamide. Apart from the thiazole ring, the final products contain many other heterocyclic rings (i.e., thiazolidine, pyrazole, pyridine, and chromene). The newly synthesized compounds were tested for their anticonvulsant effectiveness in a picrotoxin-induced convulsion model. Compound **1**, i.e., 4-(6-amino-3,5-dicyano-4-(4-methoxyphenyl)-2-oxopyridin-1(2H)-yl)-N-(thiazol-2-yl)-benzenesulfonamide, had displayed the highest anticonvulsant properties, eliminated the tonic extensor phase, and afforded 100% protection. The SAR analysis revealed that the highest activity of compound **1** might be attributed to the methoxy phenyl group attached to the pyridine ring [59]. Two sets of novel thiazole-integrated pyrrolidin-2-one and isoindoline-1,3-dione analogues were produced from aminothiazole in a multistep process. Both the sets of synthesized compounds were analyzed for anticonvulsant activity in a successful search effort. Analogue **2**, 1-(4-(naphthalen-2-yl)thiazol-2-yl)pyrrolidin-2-one, displayed the most activity, with a median anti-PTZ effective dose (ED_50_) of 18.4 mg/kg and a median toxic dose (TD_50_) of 170.2 mg/kg, resulting in a protection index (PI) of 9.2. If we closely observe the structural difference of analogue **2** with other compounds of the series, we find that it is the pyrrolidine ring attached to the thiazole moiety that may enhance the anticonvulsant activity. Naphthalene, on the other hand, had less or no role in the activity as the isoindoline derivative of the series is devoid of activity [60]. Based on cyclopentane-carbaldehyde, disubstituted 1,3-thiazoles as anti-convulsant agents were developed and screened. In the scPTZ model, compounds **3a–g** showed significant anticonvulsant action with a median effective dose of less than 20 mg/kg, which was approximately seven-times lower than the standard medication, ethosuximide. The SAR indicated that para-halogen-substituted phenyl attached to the thiazole ring is important for the activity. Furthermore, two compounds which had methyl and adamantyl substitutions also showed significant activity [61]. The anticonvulsant effect of thiazole-bearing analogues with 2,4-dioxothiazolidine and 4-thiazolidinone nuclei has been demonstrated in MES and scPTZ models. The intended molecules were produced following well-known methods such as the Knoevenagel reaction, alkylation reactions, and one-pot three-component reactions. Among the studied analogues, **4a** 5Z-(3-nitrobenzylidene)-2-(thiazol-2-ylimino)-thiazolidin-4-one, **4b** 2-[2,4-dioxo-5-(thiazol-2-ylcarbamoylmethyl)-thiazolidin-3-yl]-N-(2-trifluoromethylphenyl)acetamide, and **5** (2,4-dioxo-5-(thiazol-2-ylcarbamoylmethylene)-thiazolidin-3-yl)acetic acid ethyl ester demonstrated strong anticonvulsant action in both models. The effect of compound **4b** was similar to or higher than the reference drug sodium valproate [62].

Siddiqui et al. (2020) developed and synthesized a variety of pyridazinone-thiazole hybrids with amide linkages. Derivative **6** 2-(3-(4-chlorophenyl)-6-oxo-5,6-dihydropyridazin-1(4H)-yl)-N-(thiazol-2-yl)acetamide had shown the highest activity with a median effective dose of 24.38 mg/kg in the electroshock seizure test and 88.23 mg/kg in the chemo-shock seizure test. The SAR study of the synthesized compounds indicated that electron-withdrawing groups such as Cl, Br, and F on the phenyl ring connected at the 6th position of pyridazinone ring resulted in higher seizure protection. Further, the 4-chloropheny substitution showed the highest activity [63]. Anti-MES and anti-scPTZ responses were found in certain novel thiazole-linked (arylalkyl) azoles that were produced and tested. Analogues **7a** 1-[(2-Phenylthiazol-4-yl)methyl]-1H-1,2,4-triazole and **7b** 1-[(2-(4-Chlorophenyl)thiazol-4-yl)methyl)-1H-1,2,4-triazole provided protection ranging from 33 to 100% in both seizure models. The SAR analysis demonstrated that analogues containing the 1,2,4-triazole ring showed the highest anticonvulsant properties [64]. In a report, Łączkowski et al. presented the synthesis and anticonvulsant properties of ten new (2-(cyclopropylmethylidene) hydrazinyl) thiazoles. In the electroshock test, molecules **8a** and **8b** demonstrated anticonvulsant activity, likely due to the electron-withdrawing group attached to the para position of the phenyl ring, whereas compounds **8c** and **8d** both had substitutions in the phenyl ring with a partial electron-releasing group and demonstrated an anticonvulsant effect in PTZ-induced seizures [65]. 

### 4.2. Thiazoles as Antitumor Agents

**Figure 4:**
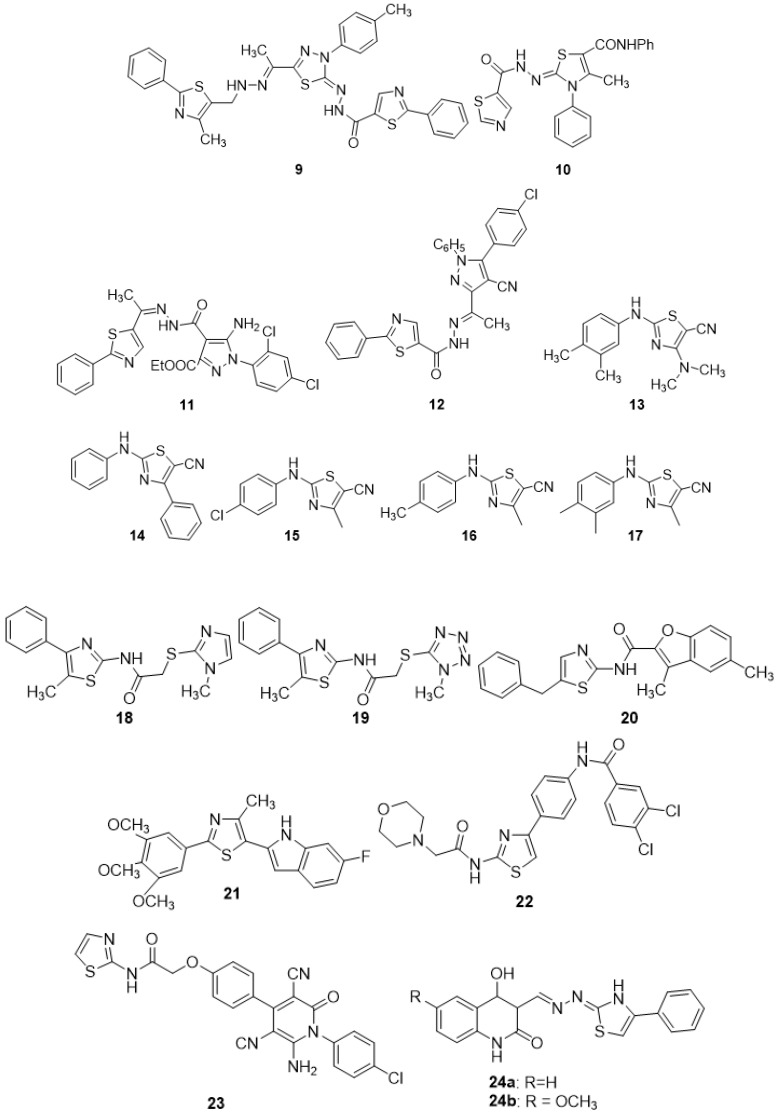

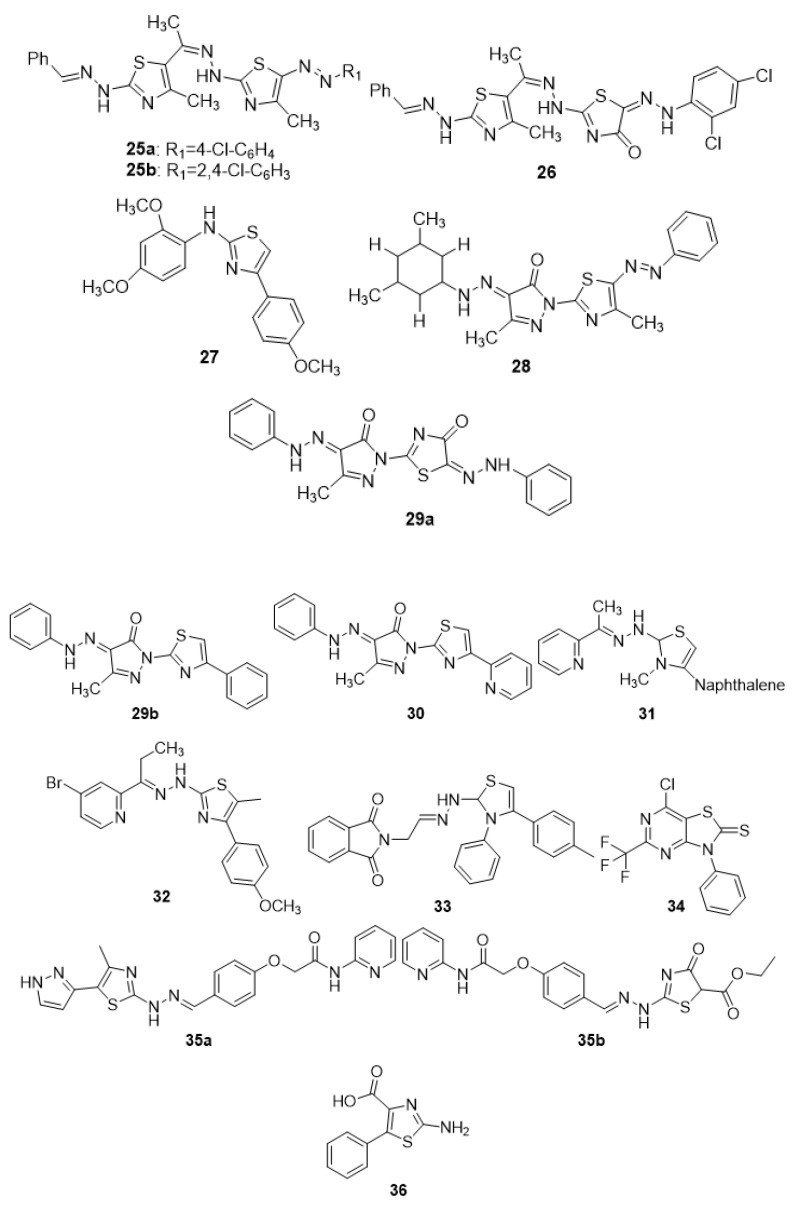
Thiazoles that have recently been designed and produced as possible anticancer agents.

In recent decades, significant headway has been made in discovering anticancer molecules, due to which several novel anticancer therapeutics have been added to the list of anticancer medicines. Thiazole, a five-membered heterocyclic motif comprising sulfur and nitrogen atoms, is a significant platform in a number of medicinally relevant molecules. Dabrafenib, dasatinib, patellamide A, ixabepilone, and epothilone are examples of clinically used anticancer medicines that contain the thiazole nucleus. Thiazole-containing compounds have recently been identified as potential inhibitors of a variety of biological targets, for example, the cell cycle (i.e., microtubular inhibitors), the cell membrane enzyme-linked receptors (polymerase inhibitors), etc. Furthermore, these molecules have demonstrated great anticancer activity and low toxicity. Figure 4 below summarizes recent research on thiazoles and explains their biological significance in anticancer medication development. Researchers may be able to use the data to develop more effective and bio-target specific anticancer medication compounds as a result of the findings.

In search of new potential antitumor agents [66], some new thiazole derivatives were synthesized using 4-methyl-2-phenylthiazole-5-carbohydrazide as synthon while keeping other structural criteria in mind. The anticancer potential of the new compounds was tested in vitro against Hepatocellular carcinoma cell lines (HepG-2) utilizing MTT assays. The most promising molecules concluded were **9** (IC_50_ = 1.61 ± 1.92 µg/mL)) and **10** (IC_50_ = 1.98 ± 1.22 µg/mL). A close look of the structure activity correlation indicated that the thiazole ring along with 1,3,4-thiadiazole ring are essential requirements for cytotoxic activity. The presence of a methyl group (electron donating group) at position 4 of the phenyl ring as observed in compound **9** increases its activity. Further, the presence of the N-phenylcarboxamide group as found in compound **10** likely augments antitumor activity. A number of novel thiazole-bearing heterocycles were produced in a separate study, employing 1,3-dipolar cycloaddition processes in the presence of chitosan-grafted-polymer (vinyl pyridine). The anti-proliferative potentials of all compounds were tested against breast cancer (MCF-7) cell lines, human hepatocellular carcinoma (HepG-2), and colorectal carcinoma (HCT-116), and the findings revealed that all compounds are effective; however, the chlorine-bearing analogues **11** and **12** were proven to be the most potent [67]. Other studies found some unique, structurally diverse thiazole analogues as potential anti-cancer drug candidates. A-431, ARPE-19, and Bcl-2-Jurkat cultures were employed to screen the newly prepared molecules. All of the analogues had undergone plenty of in vitro and in silico tests. Among the novel compounds, **13**, **14**, **15**, **16**, and **17** have significant anti-Bcl-2 Jurkat and anti-A-431 activity. Molecule **13** was equipotent against both cell lines and IC_50_ against both cell lines was less than the reference drug doxorubicin. For molecule **13**, which was significantly active in both cell lines, molecular dynamics (MD) simulations using the Bcl-2 (4IEH) protein were performed. The data revealed that **13** interacted with protein primarily through hydrophobic contacts, with only a few H-bonding interactions. SAR indicates that m, p-dimethyl substitution in the phenyl ring is important for cytotoxic activity as shown in compound **13**. The replacement of the N,N-dimethyl group with a phenyl ring in the thiazole moiety is essential for the activity (compound **14**). As observed in compounds **15** and **16**, the replacement of the N,N-dimethyl group with a simple methyl group in the thiazole ring along with the p-chloro/p-methyl group in the phenyl ring is crucial for antitumor activity. The m, p-dimethyl substitution in the phenyl ring along with the replacement of the N,N-dimethyl group with a simple methyl group in the thiazole ring was essential for cytotoxic activity (compound **17**) [68].

Evren et al. (2019) developed novel N-(5-methyl-4-phenylthiazol-2-yl)-2-substituted thioacetamides by reacting 2-chloro-N-(5-methyl-4-phenylthiazol-2-yl)acetamide with some mercapto derivatives and tested them against the NIH/3T3 mouse embryoblast cell line and A549 human lung adenocarcinoma cells for anticancer activity. Compound 19 demonstrated strong selectivity against both cell lines, with IC_50_ values of 23.30 ± 0.35 mM and >1000 mM. Compounds **18** and **19** exhibited the highest apoptosis percentage among those tested, but not as high as the standard, cisplatin. The structure activity relation indicated that tetrazole and imidazole rings are essential for activity [69]. A group of researchers synthesized four N-acylated 2-amino-5-benzyl-1, 3-thiazoles were synthesized by reacting of 2-amino-5-R-benzyl-1,3-thiazoles with acid chlorides in the presence of triethylamine in the dioxane medium. The MTT assay was used to screen the newly produced thiazoles for anticancer activity. Compound **20** was shown to be the most active for human glioblastoma U251 cells and human melanoma WM793 cells, possibly due to the presence of benzofuran ring [70]. A separate research reported a variety of indole-linked thiazoles. The synthesized products were obtained via the reaction of thioamides with 3-tosyloxypentane-2,4-dione and led to the in situ formation of 5-acetylthiazole upon which treatment with arylhydrazines in polyphosphoric acid resulted in the formation of 5-(2′-indolyl)thiazoles. These final products were tested for cytotoxicity against several cancer cell lines. Among the generated thiazoles, analogue **21** and others exhibited promising anticancer potential as well as cell line selectivity. The highest activity of this compound might be due to the presence of a methoxy group on different positions of the phenyl ring attached to the second position of the thiazole ring and fluorine substitution on the 5 position of the indole ring (IC_50_ = 10–30 µM) [71].

Zhang et al. (2018) developed and produced a variety of phenylthiazole derivatives in multi-step reactions with the starting material 2-bromo-1-(3-nitrophenyl)ethanone. The final products were then investigated for anti-proliferative activity against the cancer cell lines HT29, A549, HeLa, and Karpas299. Compound **22** had excellent growth-inhibitory effects on all four cell lines, especially the HT29 cell line. The highest activity of compound **22** may be attributed to the presence of 3,4-dichloro phenyl, which had an IC_50_ of 2.01 µM [72]. A variety of new thiazole-integrated pyridine derivatives bearing phenoxyacetamide moiety as a connecting bridge was produced in the recent investigation. The newly synthesized products were obtained by condensation of 2-(4-formylphenoxy)-N-(thiazol-2-yl)acetamide with cyanoacetic hydrazide followed by heterocyclization with acetylacetone, treatment of the produced acrylamides with malononitrile and substituted acetophenones, and heating of the generated chalcones with mononitrile in acetic acid and ammonium acetate. The antitumor activity of the newly produced thiazole pyridine hybrids was screened against PC3, MCF-7, Hep-2, and HepG2 cancer cell lines. One of the thiazole-pyridine hybrids **23** has better anti-breast cancer efficacy (IC_50_ 5.71 μM) than the standard drug 5-fluorouracil (IC_50_ 6.14 μM), which might be due to the presence of the electron-withdrawing group chlorine (Cl) attached to the 4 position of pyridine ring [73]. Mohamed and Ramadan (2020) developed phenylthiazole-incorporated quinoline derivatives via the reaction of 2-quinolone thiosemicarbazone derivatives with 2-bromoacetophenones in the presence of triethylamine at room temperature for anticancer activities. Compounds **24a** and **24b** showed remarkable activity against colon carcinoma HCT-15, whereas compound **24b** displayed an exclusively high degree of efficacy against lung cancer NCIeH322 M possibly due to the presence of the methoxy group (OCH_3_) on the quinolone ring [74]. The other recent study by Sayed et al. (2020) reported the synthesis of a range of 5-(1-(2-(thiazol-2-yl)hydrazono)ethyl)thiazole analogues by utilizing a three-component reaction using 2-(2-benzylidene hydrazinyl)-4-methylthiazole as a starting precursor. Anticancer screening against HCT-116, HepG2, and HT-29 employing the MTT colorimetric assay was carried out. Analogues **25a**, **25b**, and **26**, exhibited remarkable effectiveness against cancer cell lines compared to the standard drugs harmine and cisplatin. The analogues **25a** and **25b** displayed a high degree of efficacy possibly due to substitution on the thiazole ring with 4-cholorophenyl and 2,4- dicholorophenyl groups, respectively, and in the case of compound **26**, the 2,4- dicholorophenyl group attached to thiazole-4-one [75].

Sun et al. (2017) developed and produced a host of substituted diaryl-1,3-thiazole analogues as tubulin blockers. They synthesized these via a multi-step reaction, and the starting materials were various aniline derivatives. The bulk of the target molecules had moderate anti-proliferative action; however, compound **27** was the most effective against tubulin polymerization, and the action was likely due to the amino-linked compounds where the A ring accommodated the 2,4-dimethoxy substitutions [76]. Sayed et al. (2019) produced some novel 3-methyl-5-oxo4-(2-arylhydrazono)-4,5-dihydro-1H-pyrazole-1-carbothioamides. These new derivatives were obtained from the reaction of ethyl 3-oxo2-(2-arylhydrazono)butanoates with thiosemicarbazide. The anti-proliferative activity of products was scanned against the human liver carcinoma cell line (HepG-2) and revealed that compounds **28**, **29a**, **29b**, and **30** presented activities near the standard antiproliferative drug doxorubicin. The activity of these compounds is likely due to the presence of the phenyl ring attached with the thiazole ring via the hydrazono group [77]. New 1, 3-thiazole compounds were developed by two reaction steps: A condensation between ketones and different thiosemicarbazides, and a cyclization of the thiosemicarbazones obtained in the preceding step with different halogen-bearing acetophenones. The newly produced analogues were tested for antiproliferative activity over J774A.1 macrophages as well as HT-29 and Jurkat cells. Analogues such as **31**, **32** and **33** displayed the most effective cytotoxic and immunomodulatory potentials. Compound **33** showed its activity possibly due to the presence of naphthalene, and the rest of the compounds showed their activity perhaps due to the presence of electron-donating groups on the phenyl ring [78].

Novel thiazole pyrimidine derivatives were synthesized by the cyclization of the 4-amino-2-thioxo-2,3-dihydro-3-substituted-1,3-thiazole-5-carboxamides with trifluoroacetic anhydride during the initial phase of synthesis. The intermediates obtained after undergoing multistep reactions produced the final products. These final derivatives were evaluated for their potential anticancer activity. The antiproliferative activity evaluation was carried out on a variety of cell lines including four human cancer (A375, C32, DU145, MCF-7/WT) and two normal cell lines (CHO-K1 and HaCaT). Among the newly synthesized compounds, **34** proved to be the most active agent. The SAR analysis revealed that the presence of an electronegative Cl group is an essential requirement to elicit antiproliferative activity [79].

Pyridine-substituted thiazole hybrids were synthesized using the precursor 2-(4-((2-carbamothioylhydrazono)methyl)phenoxy)-N-(pyridin-2-yl)acetamide with various a-halogenated carbonyl compounds (namely, phenacyl bromides, ethyl bromoacetate, diethyl bromomalonate, and 3-chloropentane-2,4-dione). The cytotoxicity effect of the synthesized compounds has been studied against liver carcinoma (HepG2), laryngeal carcinoma (Hep-2), prostate cancer (PC3), breast cancer (MCF-7), and normal fibroblast cells (WI38). Among the synthesized compounds, **35a** and **35b** revealed most promising anticancer activity likely due to the presence of a thiazolin-4-one ring system, which was substituted at the fifth position with ethyl carboxylate as observed in **35a** and a pyrazole moiety at the fifth position of thiazole ring in the case of compound **35b** [80].

A series of trisubstituted thiazole derivatives were synthesized by the reaction of an appropriate aldehyde solution in dry diethyl ether with methyl dichloroacetate, which was subsequently transformed into final products. These compounds were evaluated for their carbonic anhydrase (CA)-III inhibitory activities. Among the synthesized compounds, **36** was the most potent CA-III inhibitor, and the SAR study revealed that the presence of a free amino group at the 2- position, a carboxylic acid moiety at the 4-position, and a phenyl ring at the 5-position of the thiazole scaffold were the essential requirements for anti-CA-III activity [81].

### 4.3. Thiazoles as Antimicrobial Agents

**Figure 5:**
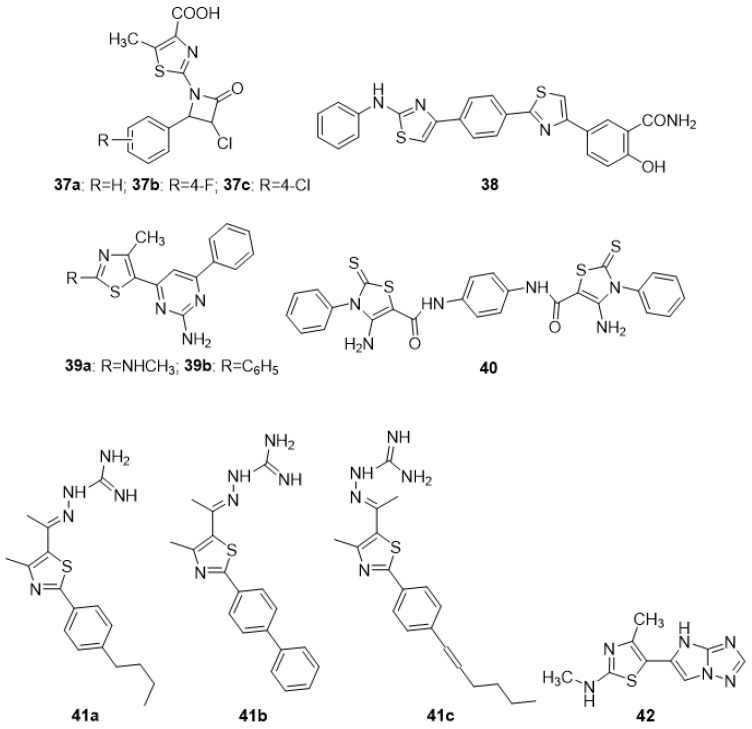

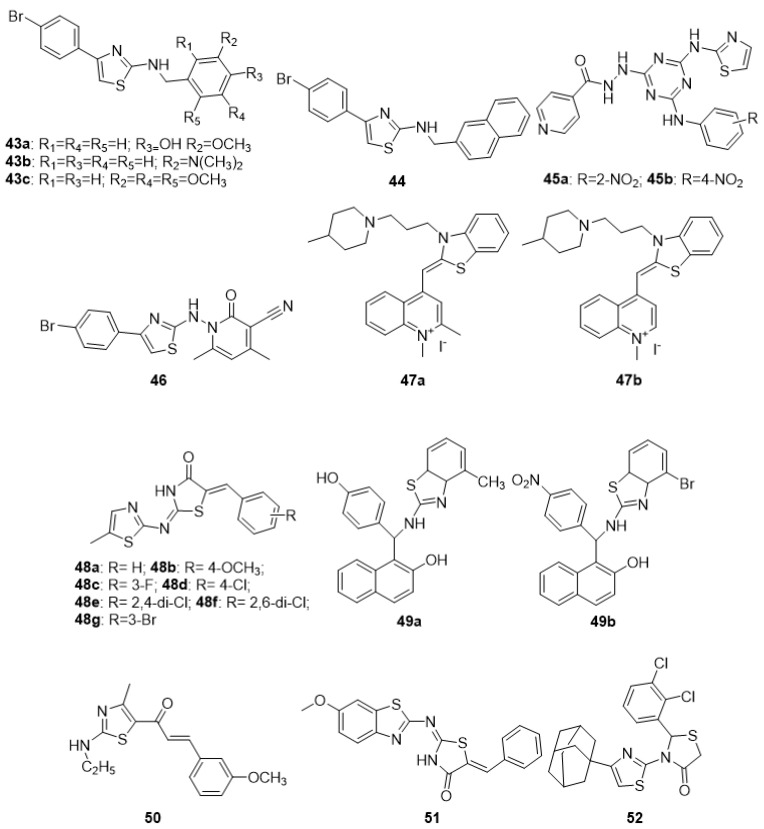
Thiazoles that were recently designed and produced as possible anti-microbial agents.

Thiazole bearing new chemical entities has contributed significantly to the progress of bio-chemical sciences over the years. Because of their unique features, they are the most-celebrated basic moiety in the drug industry. Due to their massive biological importance, scientists are working hard to develop novel, biologically active thiazole derivatives. The antibacterial potentials of several thiazoles and their derivatives were explored in this review. The current ongoing research on thiazoles as antimicrobials will assist researchers in designing and synthesizing diverse active molecules for the purpose of developing screening techniques to study their antimicrobial potentials against various pathogens and associated diseases (Figure 5).

Antibacterial activity was checked against E. coli NCTC 10418, S. aureus NCTC 65710, P. aeruginosa NCTC 10662, B. subtilis MTCC 1133, and S. typhi MTCC 1253 for an array of phenylazetidine-integrated thiazole derivatives initially synthesized via 2- chloroethyl acetoacetate. The **37a**, **37b**, and **37c** compounds in the series demonstrated the most promising action against all bacterial strains with MIC values of 6.25 µg/mL. The SAR revealed that the activity might be due to either the presence of different electron-withdrawing group at position 4 of the phenyl ring or no substitution on the phenyl ring [82]. The antimicrobial activity of numerous new thiazolylamine derivatives and thiazolylbenzamide ethers were synthesized from 4-(2- phenylamino)-thiazol-4-yl)-benzothioamide and 2-hydroxy-5-(2-(phenylamino)-thiazol-4-yl)- benzamide with several α-halo-ketones by the Hantzsch reaction and tested against various bacterial and fungus strains. Compound **38** inhibited the growth of all pathogens tested (MIC 31.25 µg/mL for Gram-positive bacteria), which might be due to the presence of a 3-carbamoyl-4-hydroxy-phenyl group [83]. Liaras et al. (2014) produced and tested a variety of amino-pyrimidine derivatives based on thiazoles for antibacterial activity. The synthesis of aimed N-phenylpyrazolines and amino-pyrimidines was afforded by heterocyclization of the corresponding chalcones with phenylhydrazine hydrochloride and guanidine hydrochloride in the presence of sodium hydroxide. Molecules **39a** and **39b** showed a better activity profile when compared to the reference drugs ampicillin and streptomycin. The SAR analysis revealed that the presence of NHCH_3_ and a phenyl group on the side chain are essential for the activity [84]. Some novel thiazole compounds have been identified as promising antibacterial agents in a recent investigation. The thiazole derivative **40** was prepared via treatment of N,N’-(1,4-phenylene)bis(2-cyanoacetamide) with elemental sulfur and phenyl isothiocyanate. Derivative **40** was found to be eequipotent to chloramphenicol against S. aureus (MIC 3.125 µg/mL) and have considerable activity against B. thuringiensis (MIC 6.25 micro-g/mL). The activity might be due to the presence of a 3-phenylthiazole-2(3H)-thione group [85]. The lead compound and three newly produced (**41a–c**) thiazole analogues exhibited potent antimicrobial activity in vivo, with similar capability as the antibiotic mupirocin, as they reduced the burden of MRSA present in skin wounds by more than 90%. The activity might be due to the presence of the phenyl ring and side chain aliphatic group at the 4-position as observed in compounds **41a** and **41c**, and an un-substituted aromatic ring as found in **41b** [86]. The antibacterial efficacy of freshly synthesized imidazotriazole-incorporated thiazoles was investigated against a spectrum of microbiological species. The synthesis was afforded by the reaction of 2-bromo-1-(4-methyl-2-(methylamino)thiazol-5-yl)ethan-1-one with heterocyclic amines, o-aminothiophenol, and thiosemicarbazone derivatives. The majority of drugs showed good to moderate activity; however, derivative **42** exceeded the activity of the standard (amphotericin B) against Staphylococcus epidermidis possibly due to the presence of an imidazotriazole ring [87]. A separate investigation demonstrated the potential antibacterial activity of several new substituted phenylthiazol-2-amine derivatives. The synthesis was carried out by p-bromoacetophenone, and thiourea was reacted in the presence of the catalyst iodine to yield the 4-(4-bromophenyl) thiazol-2-amine (intermediate). The intermediate with corresponding aromatic aldehyde yielded the target compounds. Antibacterial activity outcomes showed that compounds **43a**, **43b**, **43c**, and **44** exhibited potential antimicrobial activity comparable to that of norfloxacin as standard. The structure-based activity study revealed the presence of electron-releasing groups [OH,—OCH3] on the benzylidene portion as observed in **43a** and **43c**, the presence of an electron-withdrawing group [–N(CH3)2] on the phenyl nucleus in **43b**, and 2-OH naphthaldehyde, as found in **44,** was essential for activity [88]. The antibacterial efficiency of the target compounds was assessed using the successive dilution method against Gram -ve and +ve bacteria, which was synthesized via the reaction of 1,3,5-trichloro triazine in acetone with isoniazid (i.e., isonicotinohydrazide) at 0–5 °C in the presence of sodium hyroxide with constant stirring for 2 h during the initial phase. Analogues **45a** and **45b** were discovered to possess significantly greater antibacterial activity than the currently available antibiotics. The SAR findings attributed the activity to the NO_2_ functional group on the 2 and 4 positions of the phenyl ring [89].

A novel series of substituted thiazolyl derivatives was synthesized in good to excellent yield from the reaction of 1-(3-cyano-4,6-dimethyl-2-oxopyridin-1(2H)-yl)thiourea with 2-oxo-N’- arylpropanehydrazonoyl chloride, chloroacetone, α-bromoketones, ethyl chloroacetate, and 2,3-dichloroquinoxaline, and they were screened for their antimicrobial activities. Compound **46** shown good antibacterial activities with MIC ranging from 93.7–46.9 μg/mL; in addition, it showed good antifungal activities with MIC ranging from 7.8 to 5.8 μg/mL. The structure-based activity analysis indicated that the electron-withdrawing group (bromine) at p-position of the phenyl ring was essential to the antimicrobial activity [90].

A variety of thiazole-quinolinium derivatives with aliphatic amino and/or styrene substituents were synthesized from benzothiazolidine derivatives, and further explored for the antibacterial potential against several Gram-positive and Gram-negative bacteria. The result suggested that among the synthesized compounds, **47a** and **47b** were the most potent and effective bacteriostatic agents against multi-drug resistant bacteria. In brief, the SAR study emphasized the requirements of small groups such as CH_3_ or H at the 2-position of the quinoline fragment [91]. The novel methylthiazole-based thiazolidinones derivatives were synthesized when a solution of 2-amino-5-methylthiazole and sodium carbonate in anhydrous dimethylformamide (DMF) was added dropwise to a solution of chloroacetyl chloride in DMF, and the product obtained was exploited for multistep reactions to obtain the target compounds. All of the compounds were antibacterial, with some of them (**48a**, **48b**, **48d,** and **48f**) showing particularly good action against *E. coli* and *B. cereus*. Compound **48e** outperformed reference medications ampicillin and streptomycin in antibacterial activity against three resistant pathogens: MRSA, *P. aeruginosa*, and *E. coli*. At a concentration equal to the MIC, compounds **48c**, **48f**, and **48g** significantly reduced the development of biofilms associated with *P. aeruginosa* by more than half [92]. Novel benzothiazole derivatives were synthesized and checked for anti-microbial activity against the bacterial strain. Among the synthesized compound, the compounds **49a** and **49b** showed highest anti-microbial activity against all tested bacterial strains. The high activity of these two compounds may possibly be due to the presence of a phenyl ring substituted with hydroxy and nitro groups, and also due to the presence of dihydrobenzothiazole substituted with methyl and bromo groups [93]. Novel thiazole-based chalcones were synthesized via a Claisen–Schmidt condensation reaction. Compounds 1-(4-methyl-2- (methylamino)thiazol-5-yl)ethanone and 1-(4-methyl-2-(ethylamino)thiazol-5-yl)ethanone reacted with various aromatic aldehydes in the early phase of reactions and the products obtained had subsequently undergone multistep reactions to produce the final compounds. These newly synthesized compounds were checked for antimicrobial activity. All compounds have shown antibacterial properties better than that of ampicillin, and in many cases, better than streptomycin. The antifungal activity was also significantly high and better than the reference drugs bifonazole and ketoconazole. Among the synthesized compounds, **50** appeared to be 10- and 56-fold more potent compared to streptomycin and ampicillin, respectively. A close look at the SAR revealed that the presence of a chloro- and fluoro-substituted phenyl ring is the primary requirement [94]. Thiazole and benzothiazole derivatives, as well as thiazolidinones, were synthesized using old and classical organic synthesis methods. The antimicrobial activity was evaluated using the method of microdilution. Among the synthesized compounds, title compound **51** showed the most potent antimicrobial activity, which might be due to the presence of a methoxy group on the benzothiazole moiety [95]. Some thiazole derivatives bearing multiple ring systems were synthesized from 1-adamantyl bromomethyl ketone and thiourea as starting materials. 4-(Adamantan-1-yl) thiazol-2-amine, which was obtained in the first step, reacted with differently substituted aromatic aldehydes in the presence of mercaptoacetic acid to yield the target compounds. The newly synthesized compounds showed exceptional growth suppression of a wide range of Gram-positive bacteria, Gram-negative bacteria, and fungus when tested for antibacterial activity. The majority of the compounds outperformed the reference medications (ampicillin and streptomycin) in terms of antibacterial activity. However, the best antibacterial activity was obtained for compound **52**, with MICs from 0.9–6.25 µmol mL^−1^ × 10^−2^ and MBCs from 1.53–12.5 µmol mL^−1^ × 10^−2^. All of the analogues examined had excellent antifungal efficacy against all of the fungi that were tested. Except for *C. albicans*, compound **52** had the greatest fungistatic activity against all of the investigated fungi, with MIC values as low as 0.021–0.042 µmol mL^−1^ × 10^−2^ and MFC values as low as 0.06 µmol mL^−1^ × 10^−2^ [96]. 

### 4.4. Thiazoles as Anti-Tubercular Agents

**Figure 6:**
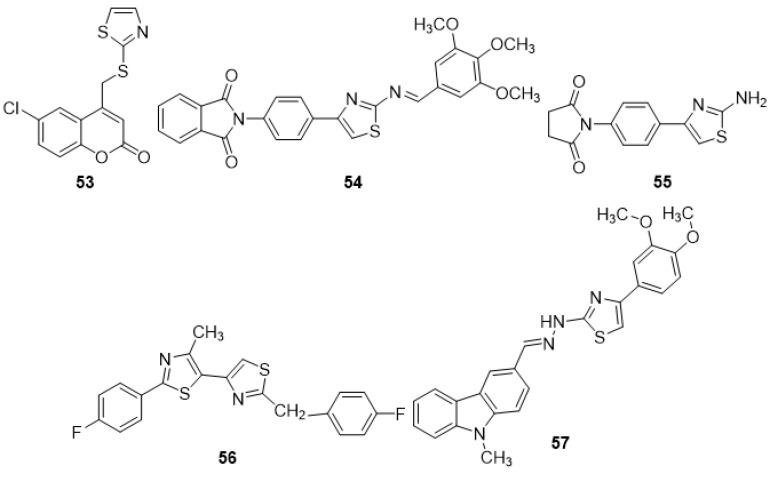

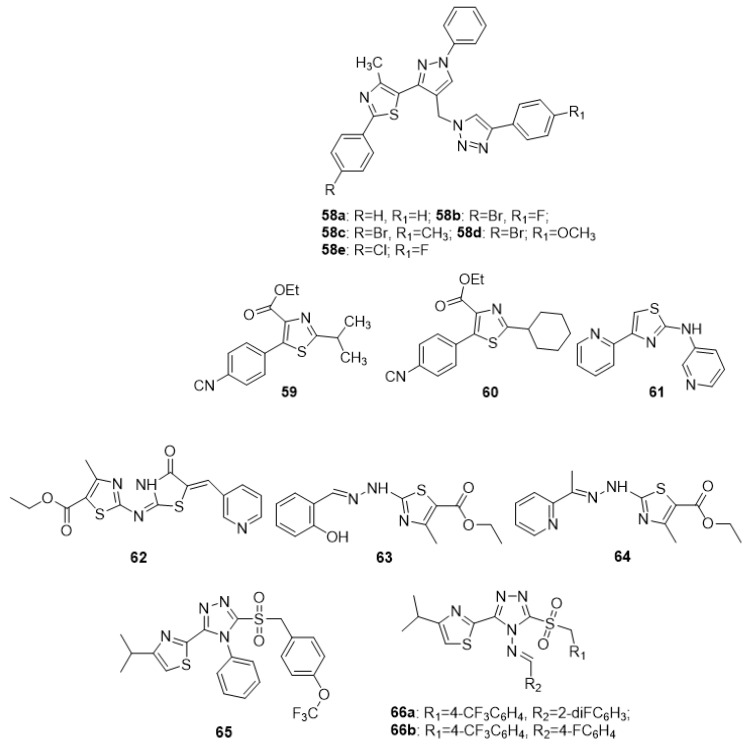

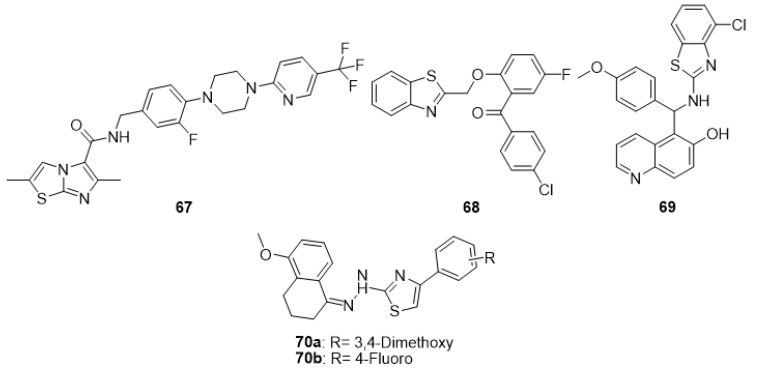
Recently designed and synthesized thiazoles as potential anti-tubercular agents.

It has been reported that tuberculosis, an infectious disease, is responsible for the deaths of millions of people worldwide each year. Many first- and second-line medications have been found to be ineffective as a result of the advent of tuberculosis resistant to multiple drugs (MDR-TB). Linezolid, bedaquiline, and pretomanid, which were recently included in TB treatment, have been linked to side effects. As a result, the introduction of safe and cost-effective anti-tubercular medications is highly required. Since the thiazole moiety is engaged in the formation of new medications used to treat a variety of diseases, it is believed that thiazole analogs may prove to be new anti-tubercular drugs or drug candidates with a high degree of safety and efficacy (Figure 6).

A series of coumarin–thiazoline hybrids was synthesized and their anti-mycobacterial activity was tested. The synthesis of target compounds was afforded by the Pechmann cyclization of phenols with 4-bromoethylacetoacetate. The condensation of 4-bromomethyl coumarin with 4,5-dihydrothiazole-2-thiol in anhydrous K_2_CO_3_ using absolute ethanol as a solvent afforded the final products 4-[(4,5-dihydro-1,3-thiazol-2-ylthio)methyl]substiuted-2H-chromen-2-one derivatives. Among the compounds examined, **53** showed excellent anti-tubercular action with an MIC of 0.09 µg/mL. The structure-based activity study indicated that the presence of a chloride ion (Cl)-linked phenyl group was essential for anti-tubercular activity [97]. Novel amino thiazoles were produced in the hopes of demonstrating activity against the Mycobacterium tuberculosis H37Rv strain. Synthesis was carried out by refluxing para amino acetophenone with maleic anhydride, phthalic anhydride, and benzoyl chloride in the presence of glacial acetic acid, which yielded 1-(4-acetyl-phenyl)-pyrrole-2, 5-dione, 2-(4-acetyl-phenyl)-isoindole-1,3-dione, and N-(4-acetyl-phenyl)-benzamide, respectively, during its initial phase of the reaction. 4-Acetyl phenyl derivatives, when reacted with thiourea and iodine, furnished respective 2-amino thiazoles. Reacting the 2-amino thiazole derivatives with their respective aldehyde substitutes furnished their Schiff bases (target compounds). The analogues **54** and **55**, which are essentially a maleic acid and thiazole ring hybrid, displayed substantial anti-tubercular activity (MIC 6.25 g/mL). The SAR indicated the presence of 3,4,5-trimethoxy substitution in compound **53** and pyrrole-2,5-dione in compound **55**, which was indispensable for the activity [98]. A number of fresh bisthiazolyl derivatives were initially synthesized by refluxing a mixture of 2-bromo-1-(2-(4-phenyl)-4-methyl thiazol-5yl)ethanone and substituted thioamide in dry ethanol. These new analogues were tested for the suppression of activity against the Mycobacterium smegmatis MC2 155 strain. At a concentration of 30 mM, analogue **56** displayed significant anti-tubercular activity. The SAR study indicated that the presence of fluoro-substituted phenyl ring is vital for suppressing the surge of *M. smegmatis* [99]. A range of substituted carbazolo-thiazoles were synthesized in good yields by a molecular hybridization approach, and in vitro anti-tubercular effectiveness was examined against the Mycobacterium H37Rv strain. Compound **57** (MIC = 21µM) displayed the most prospective anti-mycobacterial compound, possibly due to the presence of electron-donating groups (OCH3) on the phenyl ring of thiazole moiety [100]. Very recently, some promising pyrazolyl thiazole derivatives were synthesized by applying a copper-catalyzed [3 + 2] cycloaddition reaction to show activity against M. Tuberculosis H37Ra (active and dormant strain). Compounds **58a**–**e** demonstrated significant effectiveness against the Mycobacterium TB H37Ra active strain and also against the *Mycobacterium TB* H37Ra dormant strain. The structure-based activity study indicated that the presence of different R and R_1_ groups (H, Cl, Br, F, hydroxyl methyl, and methyl) attached to the phenyl ring is crucial for the activity [101].

Karale et al. (2019) prepared and investigated tri-substituted thiazoles as anti-tubercular agents. The synthesis was carried out by condensation of ethyl bromopyruvate with thioamides, which resulted in the formation of 2,4-disubstituted thiazoles. Compounds **59** and **60** suppressed dormant *Mycobacterium tuberculosis* H37Ra and *Mycobacterium tuberculosis* H37Rv strains in a very specific manner. Notably, Chinese hamster ovary (CHO) cells were not harmed by **59** or **60**. The detailed structure–activity-relationship investigation of the thiazole scaffold revealed a prerequisite for a hydrophobic substituent at C2, ester functionality at C4, and different groups with a hydrogen bond acceptor character at C5 [102]. Many substituted thiazole analogues were produced by a condensation reaction between a suitably substituted thiourea and a substituted bromoketones following tjee Hantzsch reaction. The final compounds were tested against M. tuberculosis. Molecule **61** exhibited good activity, conceivably due to the presence of amide-linked phenyl substituents at the C-2 position [103]. The new amino methyl-thiazole hybrids were synthesized by the reaction of a well-stirred hot solution of 2-[(4-methylthiazol-2-yl)amino]thiazol-4(5H)-one derivatives with sodium acetate in glacial acetic acid; thereafter, the appropriate benzaldehyde derivatives were added to obtain the final products. These newly synthesized compounds were scanned for anti-tubercular potential. The anti-tubercular potential of molecule **62** was discovered to be significant (IC_50_ = 1.56 μg/mL) and the SAR study indicated that the presence of 5-ethyl carboxylate on the thiazole ring along with 5-pyridylidene on the thiazolidinone ring was essential for anti-tubercular activity [104]. In vitro screenings were carried out to test the inhibitory ability of certain new hydrazinyl thiazole analogues against Mtb- H37Rv. A wide range of substitutions at 2, 4, and 5 positions were designed by considering the Lipinski rule. Derivatives **63** and **64** showed MICs of 12.5 µM and 25 µM against Mtb and H37Rv, respectively. The high activity of these compounds might be due to the presence of 4-methyl-2-(2-(1-(pyridin-2-yl)ethylidene) and 2-(2-(2-hydroxybenzylidene) substitutes [105]. A variety of new sulfonyl derivatives of thiazole were synthesized from the important intermediate 3-(substituted benzylthio)-5-(4-isopropylthiazol-2-yl)-4-phenyl-4H-1,2,4-triazoles. These intermediates were prepared by condensation of 4-isopropylthiazole-2-carbohydrazide with phenylisothiocyanate in the presence of ethanol. The newly produced derivatives were tested for their anti-tubercular and antibacterial capabilities. Anti-tubercular screening against Mtb H37Rv was displayed by molecules **65**, **66a**, and **66b** with higher potency when compared to the first-line antibiotic isoniazid. The SAR evaluation indicated that presence of a fluoro-substituted phenyl group was crucial for the activity (**66a** and **66b**), but in the case of **65**, the triflouoro-methoxy group was the prime requirement [106].

Nine different imidazo-thiazole-carboxamides (ITAs) analogs were synthesized when 2,6-dimethylimidazo [2,1-b] thiazole-5-carboxylic acid and (3-fluoro-4-(4-(5-(trifluoromethyl) pyridin-2-yl)piperazin-1-yl)phenyl)methanamine were dissolved in dry CH_3_CN.EDC-HCl during the initial phase of synthesis, which subsequently transformed them into final products. Among the synthesized analogs, compound **67** showed high efficacy in a chronic murine TB infection model when dosed at 200 mg/kg for 4 weeks. Upon closer observation of the structure-based activity profile, the presence of substituted fluorophenyl(trifluoromethyl) pyridinyl) piperazine was found to be essential for displaying the antimicrobial activity [107]. The new benzothiazole derivatives were synthesized and checked for their in vitro anti-tubercular activity against two tubercular strains: H37Rv (ATCC 25177) and MDR-MTB (multidrug-resistant *M. tuberculosis*, resistant to isoniazid, rifampicin, and ethambutol). Among the synthesized derivatives, compound **68** was found to exhibit significant activity with minimum inhibitory concentrations of 1 µg/mL and 2 µg/mL against H37Rv and MDR-MTB, respectively. The structural requirement likely to be highly active was the presence of chlorine and fluorine on the benzophenone moiety [108]. Some new benzothiazole derivatives were synthesized by the microwave method. The screening of test compounds for anti-TB activity was achieved by the Resazurin Microplate Assay (REMA) Plate method. It was noted that the benzothiazole (BNTZ) compound linked to a quinoline nucleus **69** exhibited remarkable anti-tubercular activity at 8 μg/mL against both the susceptible strain H37Rv and the multi-drug resistant strains of *Mycobacterium tuberculosis* [109]. Multiple series of substituted 4-arylthiazol-2-amino derivatives as modified analogues of Nitazoxanide (NTZ) were produced and tested for their inhibitory action against Mtb H37Rv in the search for new tuberculosis treatments. Among the synthesized derivatives, the two compounds **70a** (MIC = 15.28 µM) and **70b** (MIC = 17.03 µM) showed almost three times stronger Mtb growth inhibitory action than NTZ and were free of cytotoxicity (Vero CC50 of 244 and 300 µM, respectively) [110]

### 4.5. Thiazoles as Anti-Inflammatory Agents

**Figure 7:**
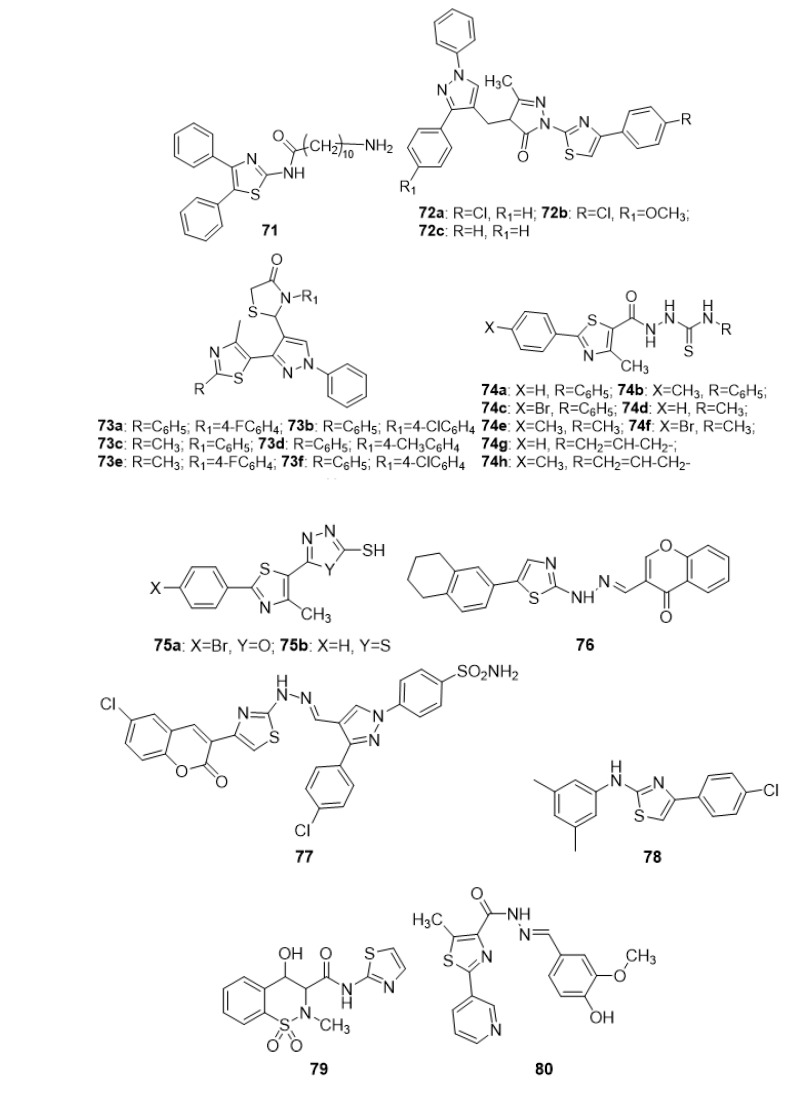

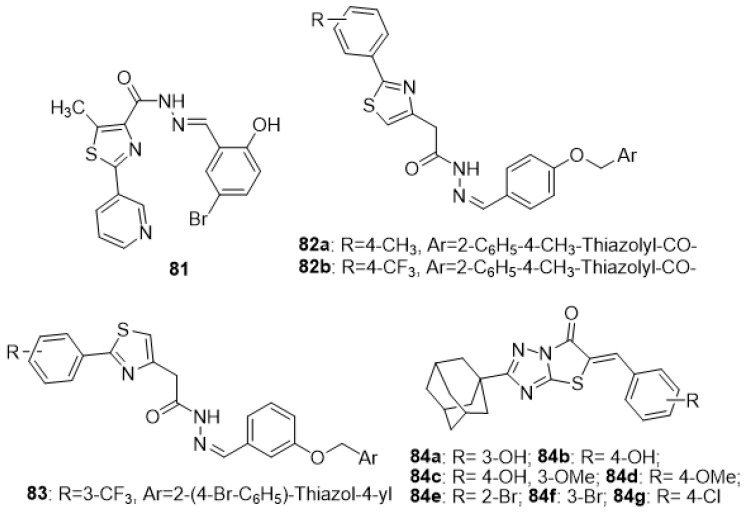
Recently designed and synthesized thiazoles as potential anti-inflammatory agents.

Inflammation is a common occurrence that has been connected to a variety of diseases and conditions, including arthritis, psoriasis, cancer, infections, asthma, and more. A cursory examination of the biochemistry of inflammation indicates that prostaglandins are the primary inflammatory marker. The bio-production of prostaglandins is taken care of by cyclooxygenase isoenzymes, also known as COX-1 and 2. Anti-inflammatory drugs belonging to the non-steroidal family, commonly called NSAIDs, inhibit cyclooxygenase isoenzymes selectively or non-selectively, and are frequently prescribed remedies for inflammation. By interfering with the arachidonic acid pathway, essentially all NSAIDs have undesirable and sometimes deadly side effects. Scientists have, in the recent past, expressed interest in developing dual COX/LOX inhibitors, which could provide a number of therapeutic advantages over standard NSAIDs, including anti-inflammatory efficacy, stomach protection, and a safer cardiovascular profile. In the quest to find dual COX/LOX inhibitors, researchers focused their attention on the thiazole moiety, which has already demonstrated a wide spectrum of pharmacological potentials and is found in various synthetic and natural drugs. Figure 7 demonstrates a collection of different thiazole derivatives, with the goal of discovering new, safe, and effective anti-inflammatory drug candidates. Abdelazeem et al. (2015) developed and evaluated a new group of diphenyl thiazole compounds linked to hydrophobic fragments via amide or urea tethers to determine their anti-inflammatory characteristics. The results indicated that compound **71** is significantly active in reducing inflamed animal paws compared to diclofenac. A quick look at the structure-based activity study revealed that the presence of decylamine was an indispensable requirement for the anti-inflammatory activity [111]. The search for novel and potential anti-inflammatory agents resulted in the synthesis of several thiazole-bearing pyrazole analogues. Phenacyl bromide, thiosemicarbazide, and ethyl acetoacetate were placed in acetic acid, heated at 60–80 °C for approximately 2–3 h during the initial stage of synthesis. The results obtained clearly showed the significance of compounds **72a**, **72b**, and **72c** as selective COX-II inhibitors. The structure-based activity analysis indicated that phenyl substituted with chlorine and methoxy groups at position 4 were the essential requirements to elicit the activity [112]. In the hopes of discovering new anti-inflammatory medicines, some new substituted pyrazoles containing thiazolyl and thiazolidinonyl moieties have been successfully and efficiently synthesized in three-step reactions using 5-acetyl thiazoles as the starting compound. According to the findings, a large proportion of compounds **73a–f** exhibited better anti-inflammatory efficacy than the reference medication Celecoxib [113]. In order to find possible inducible nitric oxide synthase (iNOS) inhibitors, novel thiazolyl carbonyl thiosemicarbazides and thiazolyl-azole compounds were designed using 2-aryl-4-methylthiazol-5-carbohydrazides and isonicotinic acid hydrazide as starting materials. For the best anti-inflammatory potential, compounds **74a–h**, **75a**, and **75b** produced superior results. Thiazole linked to substituted phenyl at position 2 and thiazole linked to substituted hydrazine-1-carbothaiomide at position 5 are important for anti-inflammatory activity in the case of compounds **74a–h** but in case of **75a** and **75b,** phenyl and bromophenyl linked to the thiazole ring at position 2 and oxadiazole and the thiadiazole ring attached to the thiazole ring were essential for the activity [114]. A different class of tetrahydronaphthalene–thiazole-coumarin multi-nucleus derivatives was synthesized for their anti-inflammatory and analgesic activity. A mixture of 1-(5-(1,2,3,4-tetrahydronaphthalen-6-yl)thiazol-2-yl)hydrazine and different aromatic aldehydes, namely, 1,3-diphenyl-1H-pyrazole-4- carboxaldehyde, 1-(3-chlorophenyl)-3-(4-methoxyphenyl)-1H-pyrazole-4-carboxaldehyde, and/or 4-oxo-4H-chromene-3-carboxaldehyde in absolute ethanol, were heated during the initial stage of reaction. In comparison to the standard drug indomethacin, the derivative **76** displayed higher anti-inflammatory and analgesic activity. The high activity might be attributed to the presence of 4H-chromen-4-one-3-yl)methylene)hydrazine [115]. In a separate study, sulfonamide-substituted coumarinylthiazoles were synthesized and tested for anti-inflammatory activity in vivo. Using the Hantzsch thiazole synthesis strategy, the present synthesis of thiazolyl hydrazinomethylidene pyrazoles consists of the condensation of appropriate 6-substituted-3-bromoacetylcoumarin with appropriate pyrazole-4-carbaldehyde thiosemicarbazone during the initial phase of reaction. Compound **77** demonstrated potent anti-inflammatory efficacy comparable to the conventional medication indomethacin. The structure-based activity analysis revealed that the presence of a substituted electron-withdrawing chlorine group is the essential requirement for eliciting the activity [116]. Anti-inflammatory investigations were conducted to examine some novel thiazole-2-amine derivatives to assess potential drug candidates. These compounds were prepared according to Hantzsch thiazole synthesis via the substitution of 2-bromoacetophenone derivatives with excess sodium thiocyanate and sequential cyclization with various aniline derivatives. Molecule **78** showed substantial anti-inflammatory potential, as reported by the research group. The SAR study indicated the presence of substituted dimethylphenyl and chlorophenyl groups as pharmacophores [117]. A single compound was synthesized by a one-pot procedure, starting from the readily available saccharine. Compound **79**, which belonged to the oxicam group and possessed high specificity for COX-2 inhibition, might possibly be due to the presence of the methyl thiazole substitute [118]. A team of researchers developed a new category of pyridine-containing thiazole moieties and studied their anti-inflammatory capabilities. Compound **80** had the highest IC_50_ value of all the chemicals examined. The in silico docking investigation demonstrated that they inhibit COX. Among the produced analogues, compound 81 emerged as a notable bioactive compound. The SAR evaluation indicated the presence of a phenyl ring substituted with OH (hydroxy) and Br (bromo) in the case of compound **81,** while in case of compound **80**, the presence of a phenyl ring substituted with methoxy (OCH_3_), and hydroxy (OH) groups were the essential requirements [119]. The synthesis and assessment of new acyl-hydrazones containing an aryl-thiazole platform as potential anti-inflammatory agents were carried out. These substances were investigated in vivo for acute experimental inflammation. Three compounds, **82a**, **82b**, and **83,** suppressed NO (nitric oxide) generation more effectively than meloxicam, the anti-inflammatory reference drug. Analyzing the chemical structures of these compounds, it was discovered that replacing the phenyl atom in position 2 of the thiazole with a bromo group in position 4 had a significant impact on the anti-inflammatory response [120].

Substituted adamantyl thiazole derivatives were synthesized when a solution of thiosemicarbazide in dry pyridine and a solution of 1-adamantylcrabonyl chloride in dry benzene were added under constant stirring at a temperature of −5 °C. The subsequent reactions produced the final products, which were screened for anti-inflammatory activity. The majority of the compounds showed a high degree of anti-inflammatory action in the PASS method. Among the synthesized compounds, **84d**, showed the highest anti-inflammatory activity among the -OH/-OMe derivatives and also displayed significant anti-COX-1 action comparable to naproxen. Some halogen derivatives also displayed a high degree of anti-inflammatory action such as **84e**, **84f**, and **84g**. These halogen derivatives were poor in anti-COX1/2 activity. In the series compound, **84b** displayed the highest COX-1 inhibitory activity (IC_50_, 30-fold lower than that of naproxen) and moderate anti-COX-2 activity. COX-2 inhibitory action was observed in the case of derivatives **84a** (3-OH) and **84c** (4-OH, 3-OMe), which was comparable to naproxen at high concentrations, but with higher IC_50_ than naproxen. A quick look at the SAR revealed that the presence of a phenyl ring with the OMe group at various positions of the phenyl ring influences the anti-inflammatory activity [121] 

### 4.6. Thiazoles as Antimalarial Agents

**Figure 8:**
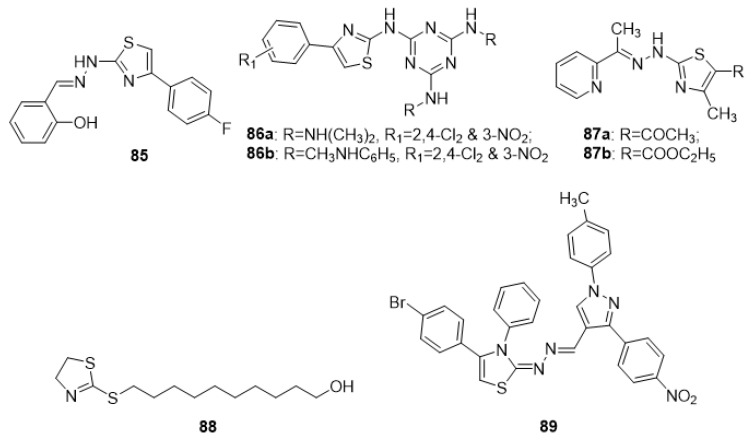

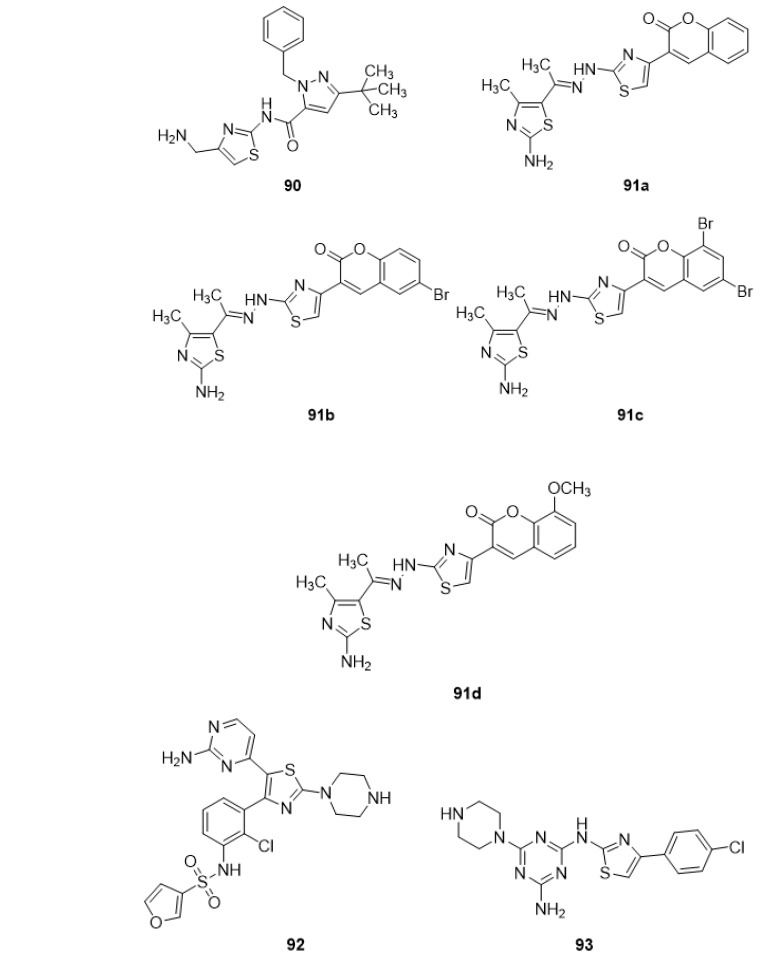

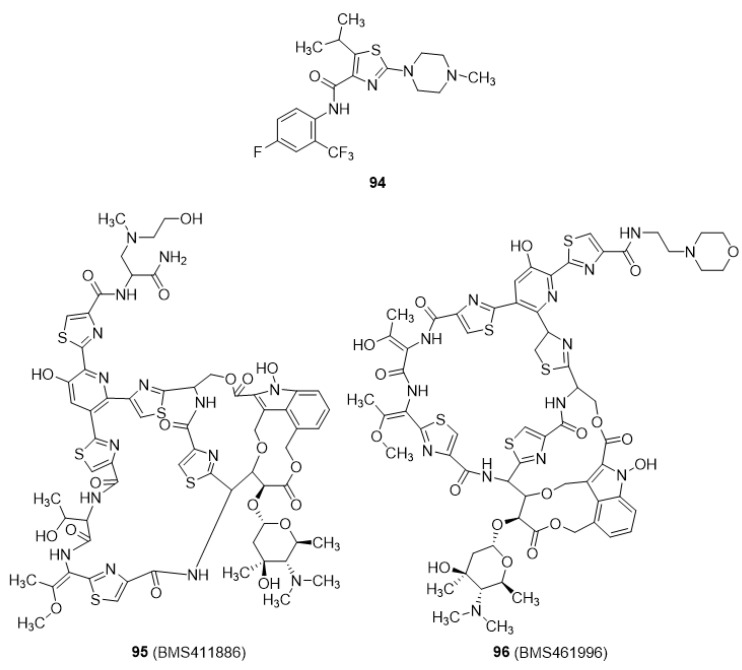
Thiazoles that have recently been developed and synthesized as possible antimalarial medicines.

Malaria is a serious worldwide health issue that results in substantial annual deaths and morbidity. The availability of alternative treatments is limited, and the advent of resistant parasite variants has posed a significant threat to malaria management. Novel antimalarial medicines with single-dose cures, broad clinical relevance, and novel mechanisms of action are immediately needed to avoid a public health crisis. Several techniques to antimalarial drug development are used, for example modifications of current agents and the generation of novel agents that operate against novel targets. Figure 8 presents the importance of the thiazole moiety in the advancement of new antimalarial agents, as reported in the literature. These agents are in the primary stage of evolution, and if research is continued, some of them may emerge as safe and effective therapeutic options for the treatment of malarial disease. Some new thiazole hydrazine analogues were initially produced when the mixture of salicylaldehyde or 5-chlorosalicylaldehyde was refluxed with thiosemicarbazide in the presence of concentrated HCl in ethanol. These analogues were scanned for antimalarial effectiveness against Plasmodium falciparum. These compounds showed some action but were not as powerful as regular quinine. One of the produced compounds, **85**, showed promising antimalarial potential against Plasmodium falciparum, with an IC_50_ similar to quinine. The SAR examination revealed that the phenyl ring substituted with hydroxy and fluorine groups at the para position is the essential requirement for eliciting the activity [122]. A library of compounds bearing thiazole-linked triazine hybrids for antimalarial activity was synthesized via a nucleophilic substitution reaction in which chlorine atoms of 1,3,5-triazine were substituted with various 2-amino-4-(substituted phenyl)thiazole and also with different aliphatic, aromatic amines. These newly produced compounds were tested over chloroquine-active (3D-7) and chloroquine-inactive (Dd-2) *P. falciparum* strains in vitro. The study concluded that analogues **86a** and **86b** were among the most potent against both sub-species of Plasmodium. The structure-based activity analysis disclosed that the presence of secondary amino substituents on the triazine ring and 2,4-dichloro and 3-nitro substituents on the phenyl ring were essential to eliciting the activity [123]. In another study, a series of hydrazinyl thiazole compounds with various substitutions were synthesized by the heterocyclization of corresponding thiosemicarbazones with aliphatic alpha-haloketones. These newly synthesized compounds were tested for their inhibitory efficacy against *Plasmodium falciparum* NF54 using an in vitro blood stage assay. The compounds **87a** and **87b** showed significant antimalarial activity with IC_50_ values of 0.725 micro-M and 0.648 micro-M, respectively. The 2-Pyridyl hydrazinyl group at the 2-position of the thiazole ring bearing an ethyl ester (COOC_2_H_5_) group at the 5- position as seen in **87b** are essential for activity. Similarly, the replacement of COOC_2_H_5_ group with COCH_3_ at the 5-position of thiazole ring, as observed in **87a**, increased the activity to a very small extent and exhibited significant antimalarial activity [124]. The synthesis of a novel thiazole-bearing derivative was carried out based on the most promising 3-alkylpyridine marine alkaloid analog. The molecule **88** was evaluated against *Plasmodium falciparum* and shown to be more effective than its antecedent (IC_50_ values of 1.55 and 14.7 µM, respectively). As it is a single molecule, the activity may be associated with the presence of a thio-linked long-chain hydrocarbon attached to the thiazole ring [125]. A new series of pyrazole-linked thiazole analogues was prepared. During the initial phase of lead synthesis, the starting compounds 1,3-diaryl-1H-pyrazole-4-carboxaldehydes were synthesized by reacting their hydrazones with the Vilsmeir–Haack reagent. The lead compound was then scanned in vivo for antimalarial efficacy over *Plasmodium berghei*-contaminated mice, with the most potent molecules being tested in vitro against the chloroquine-inactive (RKL9) Plasmodium falciparum strain. The results highlighted the greatest potency was achieved by molecules **89** along with a few others. The activity might possibly be due to the presence of a phenyl ring substituted with nitro (NO_2_) and methyl groups [126]. The thiazole analogs were synthesized via the amidation of commercially available pyrazole carboxylic acid, in the presence of the appropriate amine. Compound **90** was discovered in the Soft-Focus kinase library to be effective against chloroquine-resistant plasmodium parasites (notably K1 and NF54). The SAR analysis revealed that the presence of amino methyl thiazole substitution was necessary to display antiplasmodial activity [127]. Novel thiazolyl-hydrazonothiazole amines were synthesized by a one-pot multicomponent method using 2-amino-4-methyl-5-acetylthiazole, thiosemicarbazide, phenacyl bromide, or 3-(2-bromoacetyl)-2H-chromen-2-ones in good yield, and their antimalarial activity was tested in vitro. Four of these, **91a**, **91b**, **91c**, and **91d**, demonstrated modest efficacy against chloroquine-active and inactive *P. falciparum* strains, with half-maximal inhibitory concentration (IC_50_) values of 3.2, 2.7, 2.7, and 2.8 and 3.2, 3.2, 3.1, and 3.5 µM, respectively. The structure-based study analysis indicated that the presence of chloro, bromo, and methoxy groups’ substations on the thiazole ring was necessary to demonstrate the antimalarial activity [128]. A grading system was employed to select the sixty-six most potential PKG inhibitors. The aminopyrimidin-4-yl piperazin-1-yl)thiazole is the basic scaffold of the screening compounds. With mid-nanomolar efficacy, thiazole analogue **92** was a very potent scaffold on *P. falciparum* gamete development. The other structure that might contribute to the activity was the (chlorophenyl)furan-3-sulfonamide group [129]. Kalita et al. (2017) synthesized a number of different thiazole compounds via chloro -acetophenone, which reacted with thiourea in the presence of strong oxidizing agents such as sulfuryl chloride during the initial phase of the reaction. These compounds were tested for their antimalarial efficacy in vitro. According to the study’s findings, compound **93** is incredibly potent. The high activity might be attributed to the presence (piperazin-1-yl)-1, 3, 5-triazine-2, and 4-diamine groups on the thiazole ring [130]. Brominated thiazole was identified as a key intermediate for the preparation of the desired analogs of target compounds. The in vitro potency of a range of thiazole analogues against the chloroquine-active Plasmodium falciparum 3D7 strain was investigated. Compounds **94** showed strong antimalarial potency despite low cytotoxicity in HepG2 cell lines. The SAR analysis revealed that the presence of a phenyl ring substituted with the fluorine group at the 2 position is important for the activity [131]. Two water-soluble variants of nocathiacin **95** and **96** (BMS411886 and BMS461996) were tested for possible antimalarial efficacy against *Plasmodium falciparum* asexual blood stages. Results showed 95 (BMS461886) with significant antimalarial action and an inhibitory mean of 50% parasite growth (IC_50_) for the 3D7 strain of P. falciparum (CQ-active), 85.67 nM for P. falciparum Dd2 (accelerated drug-resistance; ARMD), and 99.44 nM for P. falciparum K1 P. Falciparum (inactive to pyrimethamine, CQ and sulfadoxine). Similar results were achieved at roughly 7-times higher IC_50_ with **95** (BMS411886) than with **96** (BMS461996) [132].

### 4.7. Thiazoles as Antiviral Agents

**Figure 9:**
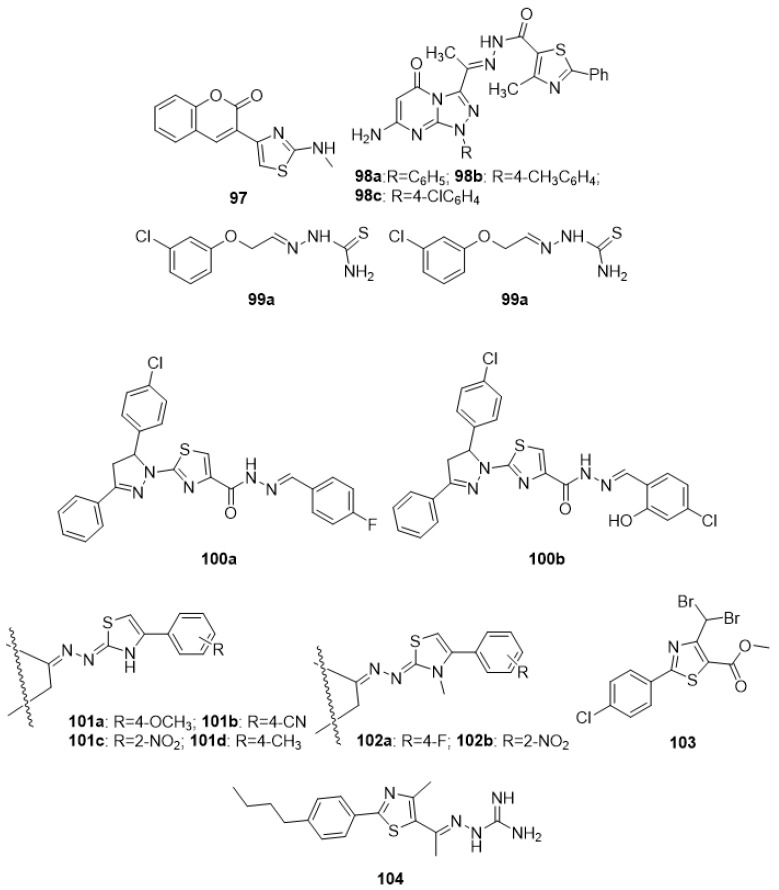

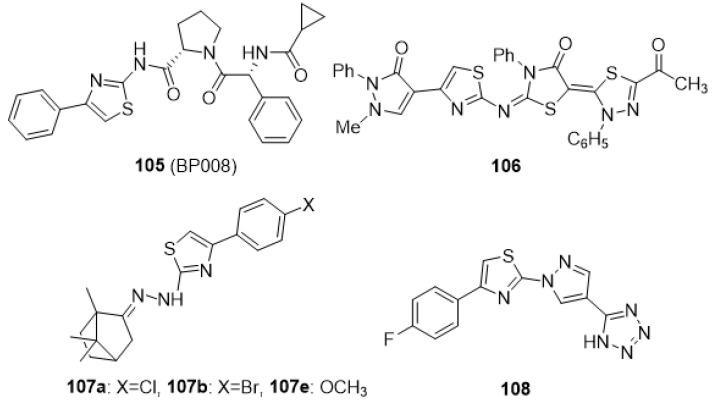
Thiazoles that have recently been designed and produced as possible antiviral drugs.

The thiazole platform is present in hundreds of drugs or developmental drugs. Despite its enormous antiviral potential, the literature contains only a few reviews on its antiviral actions. The researchers have now directed their attention to this scaffold for its antiviral activities and provided compounds with activity in the nM range. Some of the research in the previous ten years that worked to uncover the possibility of finding antiviral drugs is exemplified in Figure 9. 

Through Hantzsch cyclization of 3-(2-bromoacetyl)-2H-chrome-2- with various N-substituted thiourea/N,N-di-substituted thiourea, a variety of thiazole-containing coumarin derivatives were successfully produced as pharmacophore hybrids. These newly synthesized products were evaluated for antiviral activity. Antiviral testing results revealed that methylamino derivative **97** effectively stopped the H1N1 influenza A virus from replicating [133]. A recent study claimed to have produced several novel hydrazones with the thiazole moiety and computationally scanned them for anti-viral potential, particularly over the main protease of novel coronavirus (3CLpro). The mean binding energies of compounds **98a**, **98b**, and **98c** (−8.1 0.33, −8.0 0.35, and −8.20.21 kcal/mol, respectively) are higher than the standard Nelfinavir (-6.90.51 kcal/mol). The SAR analysis revealed that groups such as triazolo substituted phenyl, methyl, and chloro phenyl ring were essential to display anti-virus activity [134].

Pacca et al. (2017) developed and tested a variety of phthalylthiazoles for anti-SLEV and YFV activity. In a solution of 1-phenoxy-2-propanone in ethanol, thiosemicarbazide and acetic acid were added to a boiling tube during the initial phase of synthesis. The compounds were tested in vitro using flow cytometry, plaque reduction assays, cellular viability procedures, and immunofluorescence. The screening results revealed that compounds **99a** and **99b** inhibited SLEV and YFV replication more efficiently. The structure-based pharmacological analysis highlighted the presence of 3 and 3,4-di-chlorophenoxy group as essential pharmacophores to display the antivirus activity [135]. In separate research, some novel thiazoles were synthesized via treatment with 3-(4- chlorophenyl)-1-phenylprop-2-en-1-one with acetophenone and 4-chlorobenzaldehyde under basic conditions, during the initial phase of the reaction. These novel thiazoles were reported as anti-HIV-1 and HIV-2 virus employing MT-4 cells for investigations. The findings of the study showed that compounds **100a** and **100b** possess significant activity over HIV-1 with IC_50_ values of 0.50 and 0.45 µM, respectively, with selectivity indexes of 3 and 5. The SAR analysis indicated the presence of phenyl-substituted fluoro, chloro, and hydroxy groups as the primary requirements for the anti-HIV action [136]. Some researchers designed and produced a variety of steroid derivatives using thiazoline heterocycles. Generally, to a solution of DHEA in ethanol, N-substituted hydrazine carbothioamide was added at the start of the synthesis. The compounds were tested against Coxsackie Virus Type B (CVB3) and Enterovirus 71 (EV71) to explore whether they had any antiviral properties. In vitro bioassays revealed that compounds **101a**, **101c**, and **101d** had superior antiviral activity against EV71, and compounds **101a**, **101b**, **101c**, **101d**, **102a,** and **102b** had superior antiviral activity against CVB3, when compared to references ribavirin or pirodavir. The structure-based pharmacological activity analysis indicated that the presence of thiazole substituted with methyl and phenyl (substituted methoxy and nitro groups) rings played a vital role in displaying viral activity [137]. The anti-flaviviral potential of the phenylthiazole analogues has been synthesized by acid chloride, which served as a key intermediate for the replacement of the metabolically labile ester with more stable bioisosteres. Among the produced molecules, compound **103** had the highest activity, likely due to the presence of the dibromomethyl moiety present on the thiazole moiety [138]. A variety of third-generation analogues of methyl 4-(dibromomethyl)-2-(4-chlorophenyl) thiazole-5-carboxylate have been produced. Treatment of methyl alpha-chloroacetoacetate with the appropriate thioamide derivatives in absolute ethanol afforded the corresponding methyl ester derivatives. Bromination of methyl ester intermediates utilizing NBS and UV light as a free radical initiator gave dibromomethyl derivatives, which, upon further multistep reactions, produced the target compounds. These newly derived compounds were tested against the yellow fever virus in a cell-based assay, and the results were found to be promising. Compound **104** in the series was discovered to be significantly effective, which might be due to the presence of phenyl-substituted n-butane [139].

The HCV replication inhibitor molecule **105** (BP008) was synthesized as a thiazole analogue. With a 50% effective concentration and a selective index value of 4.1 ± 0.7 nM and >12.195, correspondingly, the drug suppresses the mRNA of the HCV-1b [140]. A number of thiazole-incorporated multi-ring complexes were produced and synthesized via the treatment of the 3-phenyl-1,3-thiazolidin-4-one derivative with phenylisothiocyanate in DMF, in the presence of potassium hydroxide, at room temperature, which afforded the target compounds. These compounds were tested over four viruses (influenza A (H1N1) virus, hepatitis B virus, hepatitis C virus, and poliovirus) according to a separate study. Compound **106** proved to be an effective anti-HCV agent (EC50 0.56 mM). The SAR study found that the presence of an acetyl group at C5 of thiadiazole congener provided a marked increase in the activity compared to other groups such as the ethoxycarbonyl group [141]. In this study, a range of nitrogen–sulphur-containing heterocycles, such as 1,3-thiazolidin-4-one, and thiazoles with a 1,7,7-trimethylbicycloij2.2.1]heptan scaffold, were synthesized and tested for antiviral activity. The bioassay results showed that the **107a**, **107b**, and **107c** thiazoles with a substituted benzene ring were able to inhibit vaccinia virus (VV) reproduction with IC_50_ values in the 2.4–3.7 micromolar range and moderate cytotoxicity [142]. A number of novel 2-(4-(1H-tetrazol-5-yl)-1H-pyrazol-1-yl)-4-(4-phenyl) thiazole derivatives have recently been produced. The synthesis involved a multistep reaction starting with a mixture of 1-(substituted phenylthiazol-2-yl)-1H-pyrazole-4-carbonitrile, sodium azide, and ammonium chloride in DMF, which was heated at reflux temperature for many days. These new derivatives were checked for their suppression efficacy against human PDE3B and PDE3A. Analogue **108**, with an IC_50_ of 0.24 ± 0.06 µM, inhibited PDE3A more efficiently than PDE3B (IC_50_ = 2.34 ± 0.13 µM) among the produced analogues. The SAR analysis disclosed the presence of thiazole-substituted fluoro-phenyl groups as an essential requirement to display antiviral activity [143].

### 4.8. Thiazoles as Anti-Alzheimer Agents

**Figure 10:**
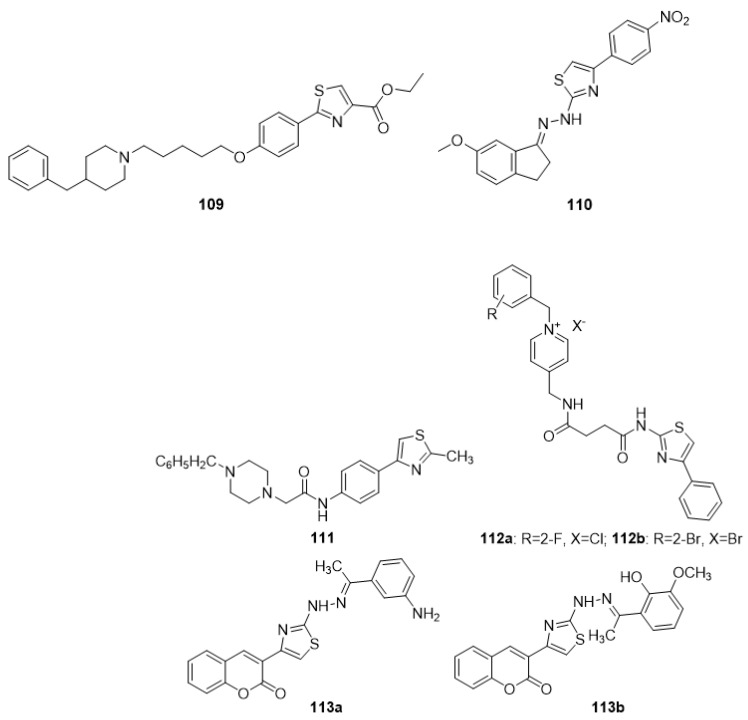

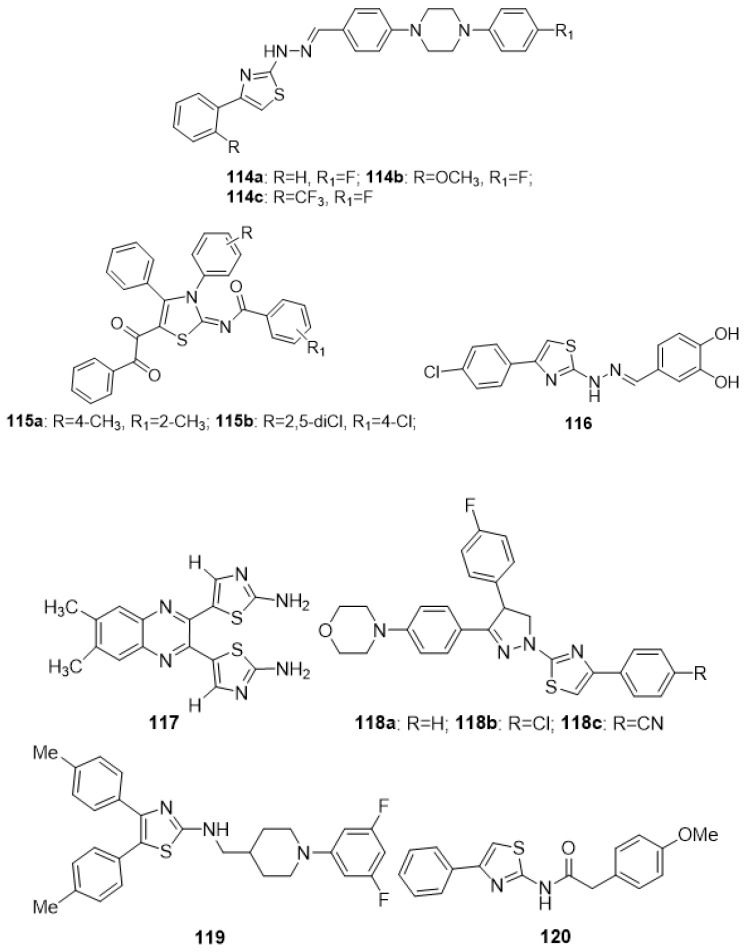

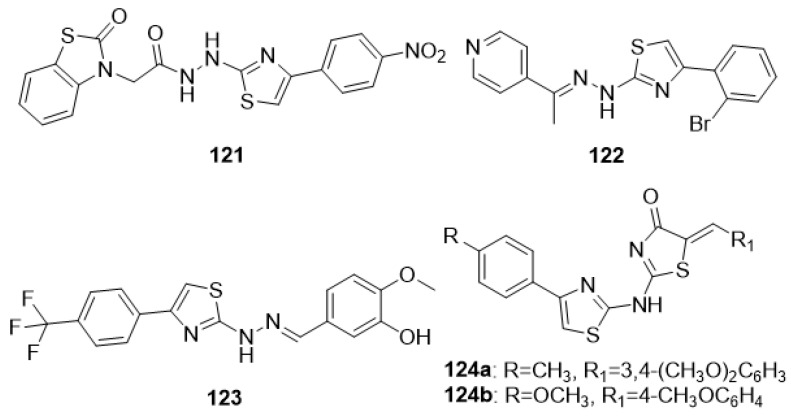
Thiazoles that have recently been planned and synthesized as possible anti-Alzheimer medicines.

Dementia is a neurological disorder that is often associated with Alzheimer’s disease (AD), although it can also occur with a variety of other CNS conditions. All treatment options should be extensively studied because the number of drugs available is limited and insufficient to provide considerable relief and enhance the sense of those who suffer from this ailment. The search for new, safe, and effective medicines is the need of the hour as old and currently available medicines do not respond in many of the cases. Thiazole-bearing new chemical entities has proven effective in the treatment of many diseases; therefore, researchers across the globe have started to focus on thiazole-bearing molecules for the evolution of new anti-Alzheimer’s drugs. Figure 10 lists some of the excellent products that have exhibited a high degree of anti-Alzheimer activity. 

Shi et al. (2017) produced new 2-phenylthiazole compounds in the hopes of finding some effective cholinesterase inhibitors. Synthesis of the first intermediate, i.e., ethyl 2-(4-hydroxyphenyl) thiazole-4-carboxylate, was achieved by combining a mixture of 4-hydroxythiobenzamide and ethyl bromopyruvate in ethanol. Thereafter, in multiple steps, the final compounds were produced. With IC_50_ values of 5.19 μM and 5.83 μM, compound **109** displayed the best acetyl cholinesterase and butyryl cholinesterase inhibition abilities. The SAR analysis indicated that the existence of (5-thiomorpholinopentyl) oxy groups was the prime requirement to exhibit anti-Alzheimer’s activity [144]. Novel thiazolyl hydrazine compounds were developed and produced based on the chemical structure of the donepezil molecule. The compounds’ inhibitory effects on beta amyloid plaque and cholinesterase (ChE) enzymes revealed that molecule **110**, with an IC_50_ value of 0.026 µM, was the most active analogue. The structure activity analysis emphasized the presence of a 4-nitrophenyl ring for the anticholinesterase and anti- beta amyloid plaque activity [145]. The anticholinesterase active novel piperazine-incorporated thiazole was synthesized when 4-aminoacetophenone reacted with acetyl chloride to obtain N-(4-acetylphenyl) acetamide to obtain the intermediates. The intermediate is subsequently used for the synthesis of target compounds. These newly derived compounds were tested on butyrylcholinesterase (BChE) and acetylcholinesterase (AChE) enzymes. The most active molecules against AChE were reported to be **111**, with an IC_50_ of 0.011 µM, whereas the IC_50_ value of the conventional medication donepezil was 0.054 µM. Analysis of the structure revealed that the presence of the piperazine-substituted benzyl group was important to exhibit activity [146]. Several new compounds with pyridinium and thiazole hybrids were synthesized using a solution of 4-oxo-4-((4-phenylthiazol-2-yl) amino)butanoic acid in dry acetonitrile with N-Ethyl-N′-(3-dimethylaminopropyl)carbodiimide (EDCI) and 1-hydroxybenzotriazole (HOBt) to obtain intermediates, which, upon further treatment, produced the target derivatives. These molecules were tested for anti-AChE and anti-Amyloid activities. Analogues **112a** and **112b** inhibited the maximum AChE at sub-micromolar concentrations (IC_50_ values of 0.40 and 0.69 µM, respectively). The SAR analysis emphasized the requirement of 2-flouro and the bromobenzyl substitute on the thiazole ring [147]. Two different derivatives were synthesized, one of which starts with the preparation of 3-(2-bromoacetyl)-2H-chromen-2-one via base-catalyzed condensation of readily available starting materials (salicylaldehyde and ethyl acetoacetate) followed by bromination. The second, the central intermediate 3-(5-thioxo-4,5-dihydro-1,3,4-oxadiazol-2-yl)-2H-chromen-2-one, was prepared by the reaction of coumarinyl hydrazide with carbon disulfide in an ethanolic solution of KOH in good yield during the starting phase of reaction. Compound **113a** displayed the most promising member of the coumarinyl thiazole group as anticholinesterase, which may possibly be due to the presence of an electron-donating amine group present at the meta-position of the aryl ring as observed in **113a** and the introduction of a hydroxy group at the ortho position and a methoxy group at the meta-position as seen in **113b**. Compound **113a** showed a median inhibitory concentration value of 0.87 ± 0.09 µM, while analogue **113b** possessed efficacy against BuChE, with a median inhibitory concentration value of 11.01 ± 3.37µM [148]. A new class of thiazole–piperazine hybrids has been produced. The ability of the produced compounds to inhibit cholinesterase enzymes was evaluated. The target compounds were synthesized via a reaction of 1-(4-fluorophenyl) piperazine and 4-fluorobenzaldehyde in the starting stage. Subsequently, the final products were synthesized. The molecules **114a**, **114b**, and **114c** inhibited the acetyl cholinesterase (AChE) enzyme significantly, likely due to the presence of thiazole-substituted phenyl, methoxy phenyl, and triflouoro-methyl phenyl groups [149]. Tetra-substituted thiazole derivatives were produced by mixing thiourea, acetophenone dissolved in DMSO in the presence of iodine, and p-toluene sulfonic acid in a refluxing mode under a nitrogen atmosphere in the early stage of the reaction, and after undergoing multiple-step reactions, the final products were obtained. These compounds were tested for biological activity against acetyl and butyl cholinesterase enzymes; compounds **115a** and **115b** displayed more potential against these enzymes. The SAR study revealed that the presence of two methyl groups on the main scaffold in the case of **115a** and three chloro groups attached with main scaffold in the case of **115b** were the essential requirements [150]. Thiazole analogues synthesized via different acetophenone/benzaldehyde were reacted and refluxed with thiosemicarbazide in methanol in the presence of catalytic glacial acetic acid during the initial stage of synthesis, and subsequently, the final products were synthesized. Among the synthesized compounds, analog **116** was found to be the most potent among the series in inhibitory activity of both acetyl cholinesterase and butyryl cholinesterase with IC_50_ values 1.59 ± 0.01 and 21.3 ± 0.50 μM, respectively. The structure activity analysis indicated the influence of hydroxyl groups on one phenyl ring and one chloro group on the other four phenyl ring [151]. As AChE inhibitors, a new group of quinoxaline-bisthiazoles targeting BACE-1 was developed by substituting 2,3-bis(bromomethyl)quinoxalines. The target products were obtained via the reaction of substituted benzene-1,2-diamines with 1,4-dibromobutane-2,3-dione in ethanol the initial starting phase, and after undergoing a series of reactions, the final products were obtained. One of the compounds, **117,** displayed BACE-1 suppression at IC_50_ of 3 ± 0.07 μM likely due to the presence of an NH_2_ functional group on the 2 position of the thiazole ring [152]. Certain new thiazolyl-pyrazolines were synthesized via molecular blending of pyrazoline and thiazole nuclei. A facile and versatile synthetic route consisting of three steps, namely the Claisen–Schmidt reaction, the formation of the 2-pyrazoline ring system, and Hantzsch thiazole synthesis, was used to prepare the compounds. Because of their considerable effects on AChE, hCA-I, and hCA-II, the in vitro and in silico tests results revealed that compounds **118a**, **118b**, and **118c** were the most promising derivatives in this series. The structure activity analysis indicated that the presence of cyanophenyl substitution may be responsible for the inhibition of hCA I (**118c**), chloro substitution exhibited the most potent and selective inhibition towards hCA II (**118b**), and without any substitution, it was defined as the most significant and selective AChE inhibitor in the case of (**118a**) [153]. Shidore et al. (2016) produced and developed a bunch of hybrid compounds by combining the pharmacophoric properties of the diarylthiazole similar to the cholinesterase inhibitor donepezil as potential therapeutics for alleviating the conditions of Alzheimer’s disease. The lead compound was prepared via the reduction of 1-substituted benzyl-N-[4,5-bis(substituted phenyl)thiazol-2- yl]piperidine-4-carboxamides in the presence of the borane-dimethyl sulphide complex during the initial phase of synthesis. The most potential compound among them (IC_50_ value: 0.30 ± 0.01 μM) for AChE and (IC_50_ value: 1.84 ± 0.03 μM) for BuChE was **119**, likely due to the presence of substituted amine and fluoro phenyl groups [154]. For prospective benefits in Alzheimer’s disease, a variety of thiazole acetamides were produced and screened for invitro butyryl cholinesterase (BChE) and acetyl cholinesterase (AChE) inhibitory activity (AD). The lead compounds were synthesized via substituted benzaldehyde, and the product was allowed to react with thiourea to afford phenyl thiazole in an efficient yield via the cyclo-condensation reaction during the initial phase of synthesis, and afterwards the final products were synthesized. Among the produced compounds, **120** was the most effective AChE inhibitor (IC_50_ = 3.14 ± 0.16 µM) with a selectivity index (SI) of 2.94 over BuChE. The SAR analysis indicated the presence of the p-methoxy group as an essential requirement [155]. The potential of several thiazole compounds to inhibit butyryl cholinesterase (BChE) and acetyl cholinesterase (AChE) was tested. These analogues were synthesized via a mixture of benzo[d]thiazol-2(3H)-one and ethyl chloroacetate in the presence of potassium carbonate in acetone, and the product obtained had undergone multistep reactions to produce the final compounds. When compared to serine (IC_50_ = 0.025 ± 0.01 µg/mL), compound **121** was shown to be the most powerful AChE inhibitor (IC_50_ = 25.5 ± 2.12 µg/mL). The activity of the analogue was most likely associated with the nitro substituent on the phenyl ring [156]. Several thiazolylhydrazone derivatives were produced and evaluated against the monoamine oxidase A and B enzymes. The lead compound was synthesized via acetylpyridine regioisomers, which reacted directly with thiosemicarbazide in ethanol in the presence of catalytic amounts of acetic acid during the early stage of synthesis. The most potent MAO-B suppressors were checked against acetylcholinesterase (AChE) thereafter. The results highlighted that analogue **122** suppresses AChE at the lowest possible concentrations likely due to the presence of a 4-acetylpyridine nucleus conferred to the hydrazothiazole derivatives [157].

New thiazolylhydrazone derivatives were designed and synthesized by reacting the substituted aldehyde derivatives with thiosemicarbazide to produce thiosemicarbazone derivatives during the initial phase of synthesis, which were subsequently converted to the final products after multistep reactions. Among the synthesized compound, **123** was found to be the most active agent in the series with an IC_50_ value of 0.028 ± 0.001 µM, which indicated an inhibition profile similar to the reference drug, donepezil. The highly active compound **123** might exhibit anticholinesterase activity due to the presence of hydroxyl and methoxy moieties at the third and fourth positions of the phenyl ring [158].

Nineteen new thiazole-based derivatives were synthesized using 4- arylthiazol-2-amines as precursors via the condensation of thiourea and the appropriate aryl methyl ketone in the presence of iodine during the initial stage of synthesis. The products obtained were subsequently transformed into the final products after undergoing multistep reactions. The in vitro assessment of their acetylcholinesterase (AChE) inhibitory activity of the synthesized compounds revealed that compounds **124a** and **124b** produced potent AChE inhibitory activity with IC_50_ values of 103.24 and 108.94 nM, respectively. The SAR analysis indicated that *p*-tolylthiazolyl and dimethoxybenzylidene were observed in **124a,** while in the case of **124b**, methoxyphenylthiazolyl and methoxybenzylidene groups were essential requirements for the activity [159].

### 4.9. Thiazoles as Anti-Diabetic Agents

**Figure 11:**
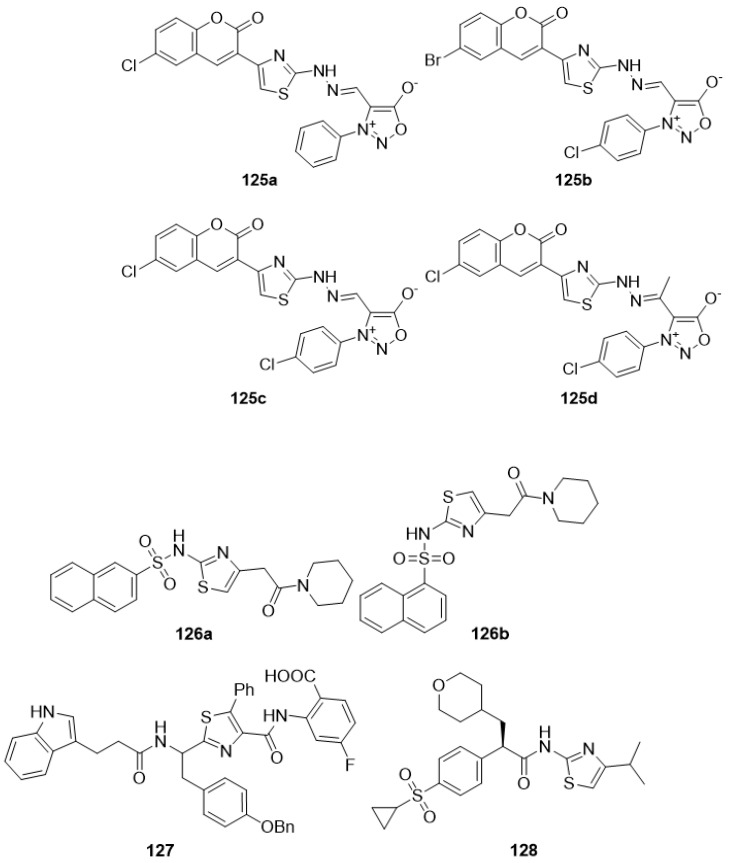

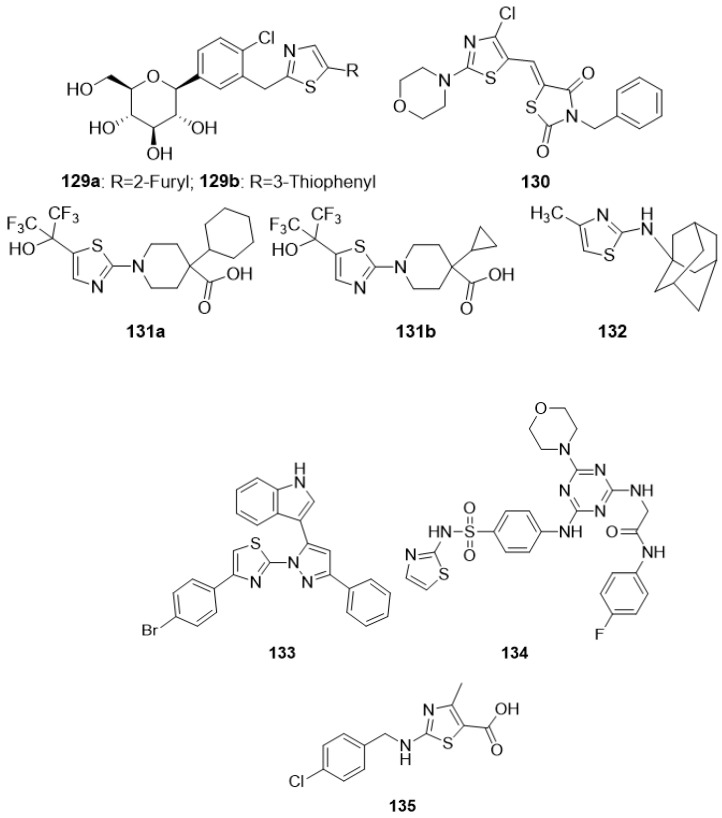
Recently designed and synthesized thiazoles as potential anti-diabetic agents.

Diabetes mellitus (DM), popularly known as diabetes, is a class of metabolic diseases characterized by a chronically high blood sugar level. Several diabetes medications or anti-diabetic medicines, including thiazolidine-diones, offer benefits and drawbacks. Due to the structural similarity to thiazolidine-diones, the thiazole moiety has huge biological potential, motivating researchers to search for novel drugs bearing a thiazole nucleus to cure diabetes mellitus. The performance of thiazoles as anti-diabetic medications is described in Figure 11.

A new thiazole-based one-pot formation of sydnones attached to coumarins was produced. Thiosemicarbazones were prepared by the reaction of 3-aryl-4-formyl/acetylsydnone with thiosemicarbazide during the initiation of synthesis, and the title molecules were screened for their α-amylase (antidiabetic) and DNA cleavage activities. In respect to the control enzyme inhibitor, the compounds **125a**, **125b**, **125c**, and **125d** had more significant inhibitory effects against α-amylase, likely due to the presence of electron-withdrawing groups in all four compounds [160]. A series of naphthalene-thiazole hybrids were produced from ethyl (2-amino-1,3-thiazol-4-yl) acetate via a coupling reaction with α or β-naphthalenesulfonyl chlorides in the presence of a catalytic amount of 4-dimethylaminopyridine and trimethylamine. These newly produced molecules were tested against the cortisone reductase (11β-HSD1) enzyme. Compounds **126a** and **126b** were shown to be considerable (55.26% and 67.03% inhibition at 10 mM, respectively) inhibitors of the enzyme. The anti-diabetic potentials of both derivatives were studied in the NIDDM rat model. Compound **126a** was solely found to be anti-diabetic at 50 mg/kg in an acute diabetic rat model. In a SAR analysis, the presence of a substituted piperidine ring was vital for the activity [161]. A novel group of PTP1B inhibitors as ethyl-phenylthiazole-carboxamide (PTA) derivatives were developed using Boc-L-Tyr(Bn)-OH, which reacted with R_4_C(O) CH(NH_2_)COOEt to provide amides during the early stage of synthesis, and subsequently, the final products were obtained. The insulin pathway was stimulated after the PTA derivative **127** reduced intracellular PTP1B. Glucose absorption was greatly boosted in cells treated with compound **127**, by virtue of the increased phosphorylation levels of Ak strain transforming (Akt) and insulin receptor b (IRb). The compound may have exhibited this effect due to the presence of flouro substitution at the 5-position of the phenyl ring along with 2-carboxyl substitution [162]. A number of thiazole-substituted arylacetamides was developed by the condensation of 4-methylsulfonyl or 4-cyclopropylsulfonyl derivatives with a corresponding aminothiazole to yield the final products. The molecule with the highest glucokinase (GK) activation efficacy was assumed to be compound **128**, which had an isopropyl group at the carbon 4 position of the thiazole ring and had a median effective dose of 0.026 µM. In the case of mice, primary cultured hepatocytes greatly boosted glucose absorption and glycogen production [163]. The new thiazole derivatives were designed and produced when carboxylic acid was coupled with 2-amino-1-(furan-2-yl)ethanone hydrochloride in the presence of EDCI, HOBt, and NMM to provide the corresponding amide, which subsequently yielded the final products. These new compounds were checked for novel sodium-glucose cotransporter-2 (SGLT2) inhibitors for biological testing. In vitro disruptive action against SGLT2 can be seen by compounds **129a** and **129b** (with IC_50_ = 0.720 nM for **129a** and IC_50_ = 0.772 nM for **129b**). The substitution of phenyl with 2-furyl or 3-thiophenyl dramatically improved the inhibitory action [164]. Morpholino thiazolyl-2,4-thiazolidinediones produced via 2,4-dichlorothiazole-5-carbaldehyde was obtained with 2,4-TZD and N,N-dimethylformamide in phosphoryl chloride during the early stage of synthesis and tested in vitro for their ability to stimulate insulin release and glucose uptake. The most effective compound in this family is **130.** The SAR analysis revealed that the presence of a substituted benzyl group at the C-3 position is essential for activity [165]. A new class of thiazole-substituted hexafluoropropanols was produced. Starting from 2- amino-thiazole, condensation with hexafluoroacetone hydrate gave rise to 2-(2-aminothiazol-5-yl)-1,1,1,3,3,3-hexafluoropropan-2-ol and the condensation product afterward yielded the final products. These newly synthesized products were tested as MCD inhibitors. In obese mice, key analogues reduced the weight of the body and the amount of food they consumed in a dose-dependent way. Treatment with these molecules for a brief span of time reduced increased blood glucose levels in a murine model of type II diabetes, according to the findings. The molecules **131a** and **131b** act as potent inhibitors of malonyl-CoA decarboxylase (MCD) likely due to the replacement of an aryl group with an alkyl group in this series [166]. In a streptozotocin-induced diabetic mellitus model, a novel adamantyl thiazole derivative, **132**, was synthesized when chloroacetone was added to a solution of *N*-adamantyl thiourea in acetone during the initiation of synthesis, and later on, the final products were synthesized. In the study, the researchers discovered that treatment with compound **132** reduced the amounts of critical glucose metabolism enzymes in liver tissues to near-normal levels, including glycogen synthase and phosphorylase, fructose-1,6-bisphosphatase, glucose-6-phosphatase, and glucokinase. The structure-based activity analysis emphasized the substitute as an essential requirement for exhibiting the activity [167]. A new group of substituted pyrazoles including indole and thiazole motifs was prepared, starting with indole-3-carbaldehyde, which was condensed with acetophenone in basic conditions to afford chalcone (1, 3-(1*H*-indol-3-yl)-1-phenylprop-2-en-1-one), which later yielded the final products. These analogues were tested for antihyperglycemic activity against the α-glucosidase and α-amylase enzymes. Compound **133** (IC_50_ = 236.1 μg/mL) demonstrated the best antihyperglycemic effect in comparison to the conventional drug (acarbose, IC_50_ = 171.8 μg/mL) likely due to the presence of bromo substituent on the phenyl ring of the thiazole moiety [168]. Hybrid molecules containing thiazole-linked triazinesulphonamide were synthesized. Initially, during the synthesis, the commercially available starting material cyanuric chloride was allowed to react with ammonia gas to afford 4,6-dichloro-1,3,5-triazin-2-amine, which. In a multistep reaction, yielded the final products. According to the findings, analogue **134** was shown to be a more potent inhibitor of DPP-4 (2.32 nM) than the standard alogliptin (3.56 nM). This high activity might possibly be due to the addition of a fluoro group in place of the chloro group at position 4 of the phenyl ring [169]. A novel thiazole derivative 2-[(4-chlorobenzyl) amino]-4-methyl-1,3-thiazole-5-carboxylic acid (BAC) was produced when substituted ethyl 2-(benzylamino)-4-methyl-1,3-thiazole-5- carboxylate and potassium carbonate were added to a mixture of methanol and water, which, upon further treatment, yielded the final compounds. The new analogues were estimated in a neonatal model of NIDDM (non-insulin-dependent diabetes mellitus) rats induced by streptozotocin (STZ). The findings of the study highlight that compound **135** is effective in reducing glucose levels in blood. The structure-based activity analysis indicated that the presence of a substituted chlorobenzyl amino group was essential for activity [170].

### 4.10. Thiazoles as A1-Receptor Antagonist

**Figure 12:**
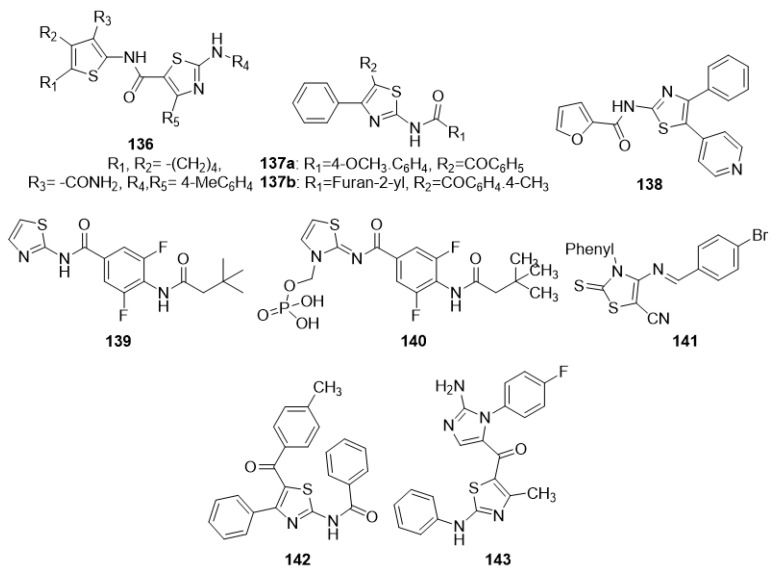
Recently designed and synthesized thiazoles as potential A1-receptor antagonist.

Adenosine receptors are classified into three types: A1, A2, and A3 receptors. Adenosine A1R activators may be helpful in conditions such as stroke, migraine, epilepsy, and pain, whereas antagonists may be beneficial in conditions such as cognitive impairments and edema. Various research groups have investigated thiazole compounds and their analogues as powerful adenosine receptor ligands, as demonstrated in Figure 12.

Unique thiazole-linked thiophene conjugates were developed to act as adenosine receptor blockers. The respective starting derivatives of 2-chloroacetamidothiophene and amidinothiourea were prepared separately. The reaction between 2-chloroacetamidothiophenes and amidinothioureas was carried out to obtain the thiazole-thiophene conjugate compounds in an early stage of synthesis. The majority of the compounds demonstrated strong affinity for the adenosine receptor subtypes A1, A2A, and A3. With an A3 Ki value of 0.33 µM and selectivity ratios of > 90, of all the compounds examined, **136** showed the most powerful and selective inhibitory molecules. The SAR analysis highlighted that the presence of a 4-methylphenyl group at 2 and 4 positions was essential for the activity [171]. In other research, A1 and A3 adenosine receptor antagonists, including dual-target molecules, were made by inserting C5 and N2 into the flexible 2-amino-4-phenylthiazole scaffold. The lead compound was synthesized via 4-methoxybenzoyl chloride, benzoyl chloride, or furan-2-carbonyl chloride. These compounds were reacted with ammonium thiocyanate in acetone to afford the intermediate isothiocyanates. Furthermore, Ki values for the dual A1/A3 antagonists **137a** and **137b** ranged from 8 to 42 nM. The most potent inhibitor was **137b,** likely due to the presence of the combination of 4-methylbenzoyl in position 5 with a furan-2-carboxamide in position 2 of the thiazole [172]. A collection of furamide compounds were synthesized using the mixture of benzoyl or 2-furanoyl chloride, benzene, and tetra butyl ammonium bromide, which was stirred at room temperature during the early stage of synthesis and examined in radio ligand binding experiments at adenosine receptor subtypes with the objective of obtaining selective and strong AR ligands. Compound **138** was shown to be the best in this study, with sub nanomolar concentration affinity for all AR subtypes. The SAR analysis indicated the presence of the 4-pyridyl substitute as the essential requirement [173]. New derivatives of thiazole were obtained based on the precursors istradefylline and preladenant. The anti-hA2A receptor activity of a variety of thiazole benzamides was tested. The efficacy of compounds **139** and **140** appeared encouraging, possibly due to the presence of flourine substituents at both 3 and 5 positions of the phenyl ring [174]. The compounds (4*E*)-4-(4-substitutedbenzylideneamino)-3-substituted-2,3-dihydro-2-thioxothiazole-5-carbonitrile were synthesized using an equimolar mixture of 4-amino-*N*3-substituted-2, 3-dihydro-2-thioxothiazole-5-carbonitrile, and *p*-substituted benzaldehyde with a small amount of AlCl_3_ in chloroform during the early stage of the reaction. The strong interaction of several experimental drugs with the A2 adenosine receptor in docking studies was corroborated by the high binding energy to A2AR in HEK293T cells employing a radio-ligand-binding test. Compound **141** showed very strong A2AR selectivity in comparison to the conventional A2AR antagonist SCH58261. The structure-based activity analysis indicated the presence of only a mesomeric effect in *N3*- phenyl groups and a bromo group substituted at the 4 position of the other phenyl ring [175]. To generate robust A1-selective antagonists, a set of 2-amino-5-benzoyl-4-phenylthiazole analogues were produced and studied. Diethylamine was reacted with benzonitrile in the presence of aluminum chloride to form *N,N*-diethylbenzimidamide, which, after undergoing a series of reactions, yielded the final derivatives. Compound **142** could be used as a lead structure for the creation of second-generation non-xanthine derived A1 antagonists with innovative therapeutic potential. The high activity might possibly be due to the presence of a phenyl ring substituted with a methyl group [176].

Pandya et al. (2018) produced a small-molecular virtual library of 2-amino-imidazolyl thiazole analogues using a 2-chlorotrityl resin. The chemical library was subjected to exhaustive screening with various subcategories of the human adenosine receptor. Compound **143** was the most potent and specific non-xanthine human adenosine A3 receptor antagonist in this class. The SAR analysis revealed that the phenyl ring substituted with a fluoro group is essential to exhibiting anti-A1 receptor activity [177].

### 4.11. Thiazoles as Bioactive (Antioxidant, Cardiotonic, Antithrombotic, Insecticide, and Anti-Repellent) Agents

**Figure 13:**
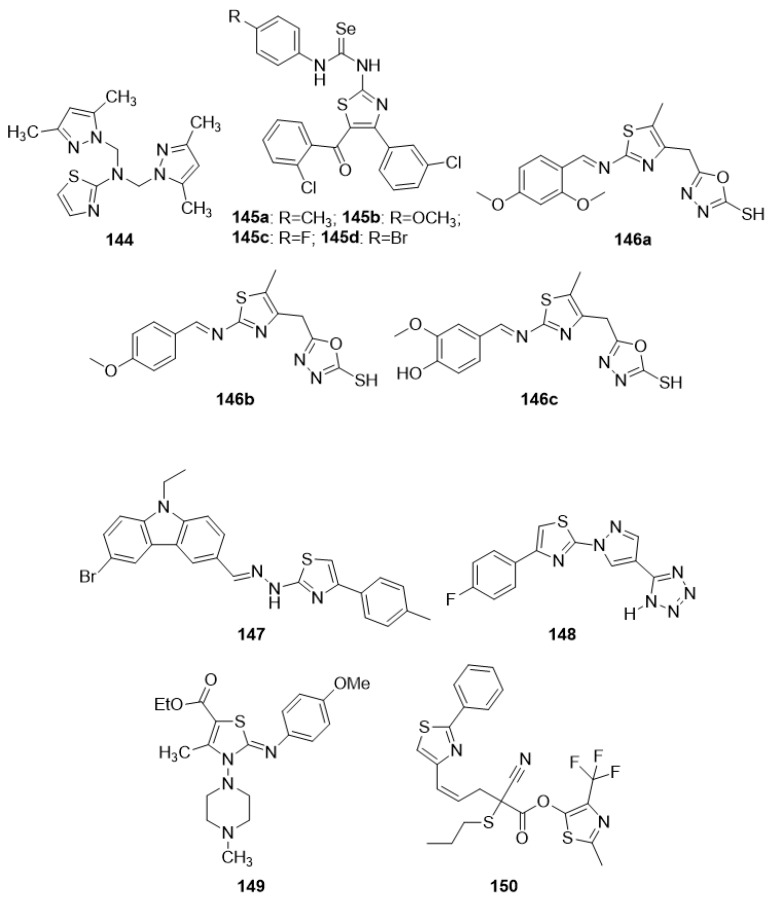

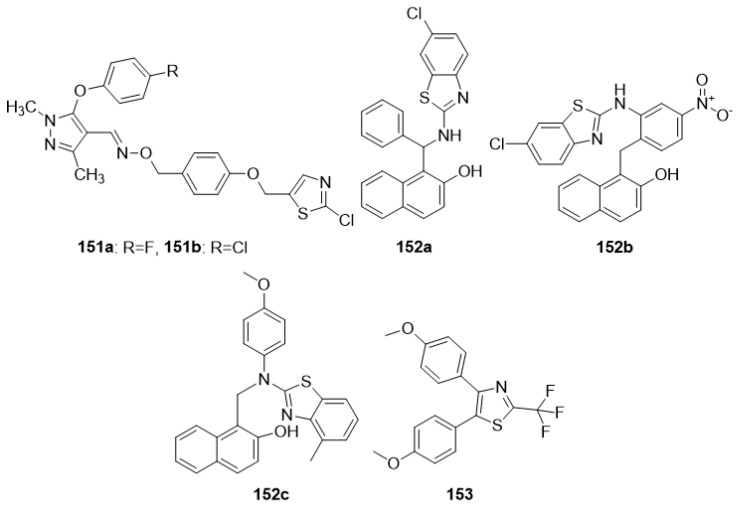
Recently designed and synthesized thiazoles as potential other bioactive agents.

Apart from the primary functions discussed previously, thiazole compounds have acted on a range of targets and exhibited a wide diversity of biological activities. In Figure 13, several examples of thiazole derivatives with numerous biological activities are included.

The thiazole, pyrazole, and pyridine nuclei were linked to generate new heterocyclic compounds based on the condensation reaction between 1,3-thiazole or aminopyridine derivatives and 1H-pyrazole,3,5- dimethyl-1H-pyrazole, or 1,2,4-triazole. Their anti-oxidant (AO) property was assessed using a DPPH scavenging test. The molecule **144** demonstrated the best antioxidant activity, with an IC_50_ of 4.67 µg/mL. The SAR study showed that the presence of substituted thiazol-2- amine was indispensable for displaying the activity [178]. A novel group of selenourea bearing a thiazole ring was designed and synthesized by the nucleophilic addition reaction of (2-amino-4-(3- chlorophenyl) thiazol-5-yl)(2-chlorophenyl)methanone with various substituted isocyanates/isothiocyanates/isoselenocynates in acetone, with a catalytic amount of sodium hydroxide at room temperature. DPPH, NO, and H_2_O_2_ (hydrogen peroxide) radical scavenging screenings were employed to test the antioxidant characteristics of each molecule in vitro. The selenourea compounds **145a**, **145b**, **145c**, and **145d** were proven to have considerable antioxidant activity (AO) and lower IC_50_ values than the reference drugs. In a preliminary analysis of the structure–activity relationship, it was discovered that compounds with selenourea functionality and a halogen group had higher activity than the standards [179]. A novel group of 5-(2-amino-5-methylthiazol-4-yl)-1,3,4-oxadiazole-2-thiol analogues have been produced via the reaction of ethyl 4-bromo-3-oxopentanoate with thiourea in EtOH, which resulted in the formation of an ester compound during the early stage of synthesis. By employing DPPH, hydroxyl, NO, and superoxide radical scavenging tests, the compound’s antioxidant properties were investigated. The radical scavenging properties of compounds **146a**, **146b**, and **146c** have been demonstrated, possibly due to the presence of an electron-donating substituent on substituted aldehydes [180]. Tran et al. (2014) have produced a unique set of disubstituted thiazole derivatives based on carbazoles when 2-((6-bromo-9-ethyl-9H carbazol-3-yl)methylene)-hydrazinecarbothioamide was synthesized by the condensation of 6-bromo-9-ethyl-9H-carbazole-3-carbaldehyde with thiosemicarbazide in ethanol in the presence of a catalytic amount of acetic acid in the initial step of synthesis, and the product obtained was subsequently converted into the final products. The anti-oxidant (AO) attributes of these molecules were tested. Compound **147** had higher antioxidant properties than the standard BHT. The activity of compound **147** might be due to the presence of the p-tolylthiazole substitute [181]. A broad range of thiazole compounds containing pyrazole and tetrazole were initially produced with the synthesis of compound 4-(4-fluorophenyl)-2-(1H-pyrazol-1-yl) thiazole, and after a series of subsequent reactions, the final products were obtained. These newly derived compounds were put to the test for inhibitory and cardiotonic activities against human PDE3A and PDE3B. Compound **148** was very active among all synthesized compounds, likely due to the presence of a flouro group on the para position in the phenyl ring [143]. Several synthetic aryliminothiazoles were studied for their cardio-protective potentials and synthesized by the Hantzsch reaction of thioureas and 3-chloropentane-2,4-dione or ethyl-2-chloro-3-oxobutanoate. Compound **149** was recognized to have excellent cardio protective effects. In isolated rings of the thoracic rat aorta, the chemical inhibited the development of constrictor responses, and increased the L-carnitine and meldonium activities by 18.2% and 12.9%, respectively. The structure-based activity analysis revealed the presence of substituents at positions 3 and 5 of the thiazole group [182]. A novel group of thiazole acrylonitrile derivatives was produced by reacting 3-hydroxy-3-(2-chloro-4-(trifluoromethyl) thiazole-5-yl)-2-(2-phenylthiazole-4-yl)-acrylonitrile with the desired acid chlorides at room temperature in the presence of triethylamine. The bioassay outcomes revealed that few title compounds were 100% lethal to Aphis fabae at 50 mg/L. The component **150**, in particular, proved to be the most powerful insecticide likely due to the presence of the propylthio-propanoate substitute [183].

A group of researchers produced a category of pyrazoleoximes with a modified thiazole ring using 2-chloro-5-chloromethylthiazole as the starting material. The bioassay results revealed that few compounds had promising insecticidal effects. Compounds 151a and 151b were more active against *Tetranychus cinnabarinus* and *Plutella xylostella* in regard to other derivatives. The SAR analysis indicated that the presence of fluoro and chloro substitutions on the phenyl ring were the essential requirements for eliciting the insecticidal activity [184].

The new substituted benzothiazole analogues were synthesized via a three-component condensation reaction. The substituted arylaldehyde, 2-amino-6-halo/4-methyl-benzo[d]thiazole, and 2-naphthol or 6-hydroxy quinoline reacted in the presence of NaCl in water via the microwave method. The final compounds obtained out of these reactions were checked for anti-repellent activity against Anopheles arabiensis mosquitoes. Among these analogous, **152a**, **152b,** and **152c** exhibited the highest repellent activity comparable to the positive control DEET. The structure-based activity analysis indicated that the presence of substituted phenyl, nitrophenyl, and methoxyphenyl groups played a vital role in demonstrating the mosquito repellant activity of the mentioned compounds [185]. In vitro evaluation, utilizing platelets from at least six species, including man, demonstrated a thiazole derivative called 4,5-bis(p-methoxyphenyl)-2-(trifluoromethyl)-thiazole **153** to be a potent suppressor of collagen-induced platelet aggregation. At a concentration of 1 ng/mL, it was effective in human platelet-rich plasma. While its antiplatelet activity was larger than that of flurbiprofen, its cyclooxygenase activity was equal to that of flurbiprofen. In the hind-paw edema test, the thiazole demonstrated less anti-inflammatory efficacy than flurbiprofen. Following oral treatment of the thiazole derivative, platelet aggregation was reduced in five laboratory species. It displayed this activity in guinea pigs when treated with 0.5 mg/kg of the compound. The median lethal dose in mice exceeded 1000 mg/kg (i.p.). This antithrombotic compound, which was developed through a methodical drug development procedure, has a great deal of potential [186].

## 5. Conclusions

In this review article, the chronology of thiazole-derived compounds has been organized and orderly presented. This review is divided into three major sections: (1) Thiazoles as treatment drugs, (2) thiazoles in clinical trials, and (3) thiazoles in preclinical and developmental stages. The preclinical investigation section included research since 2010, with some exceptions. The preclinical investigation section listed 11 subsections: Thiazole as anticonvulsant, antimicrobial, antitubercular, anti-inflammatory, antimalarial, antiviral, anti-Alzheimer, antidiabetics, anti-A1 receptor, and bioactive agents. Numerous thiazole compounds included in this review appeared to be more potent than reference medications, suggesting that they are excellent candidates for further modification and development, which may lead to future drugs.

The list of prescription drugs bearing thiazole ring is long, and only a few have been presented in Figure 1 and Figure 2. 

The second important section in this review is thiazoles in the stages of clinical trials. Some thiazole-bearing drugs have recently completed clinical trials and been approved by the USFDA. For example, mirabegron has recently been approved to treat overactive bladder. Ziritaxestat (GLPG1690), an autotaxin inhibitor, was considered a potential therapeutic candidate for the treatment of idiopathic pulmonary fibrosis. A recent report from “clinical trials.gov” showed that the Ziritaxestat phase 3 trial had been discontinued due to the benefit–risk profile. SRT2104 (GSK2245840) is a selective sirtuin 1(SIRT1) activator engaged in energy homeostasis management. This drug candidate is being investigated for the clinical use of atrophy, sepsis, psoriasis, type 2 diabetes mellitus, and muscular dystrophy. The clinical trial results (NCT01154101 and NCT01031108) and a good number of high-quality research indicated the potential of SIRT1 in the treatment of many diseases. Similarly, IRAK inhibitor 6 is a selective renal carcinoma antigen NY REN 64 blocker that is possibly used to manage bone degradation and rheumatoid arthritis-induced joint inflammation. This drug is associated with several clinical trials (NCT04440410 and NCT04815811) for other investigations. The other drugs, for example Fatostatin, WEHI-539, O4I2, TP0427736, SC75741, SRT3025, and UM-164, are being optimized for clinical trials.

The third preclinical and development stage has described many promising agents, which can further be investigated for their potential in clinical translation. For example, in the subsection “thiazole as anticonvulsants,” many agents showed potential better than the standard drugs. Compounds **3a**, **3b**, **3c**, **3d**, **3e**, **3f**, and **3g** showed significant anticonvulsant action with a median effective dose of less than 20 mg/kg, which was approximately seven-times lower than the standard medication, ethosuximide. In other study analogs, **4a**, **4b**, and **5** demonstrated strong anticonvulsant action in both models. The effect of compound **4b** was similar to or higher than the reference drug sodium valproate. 

In “thiazoles as antitumor agents,” some thiazole-bearing compounds showed excellent antitumor activities. For example, studies found some unique structurally diverse thiazole analogs as potential anti-cancer drug candidates. A-431, ARPE-19, and Bcl-2-Jurkat cultures were employed to screen the newly prepared thiazole derivatives. Analogs **13**, **14**, **15**, **16**, and **17** have significant anti-Bcl-2 Jurkat and anti-A-431 activity. Molecule 13 was equipotent against both cell lines, with IC_50_ against both cell lines being lesser than the reference drug doxorubicin. In another study, one of the thiazole-pyridine hybrids **23** showed better anti-breast cancer efficacy (IC_50_ 5.71 μM) than the standard drug 5-fluorouracil (IC_50_ 6.14 μM). Some 5-(1-(2-(thiazol-2-yl)hydrazono)ethyl)thiazole analogs, such as **25a**, **25b**, and **26,** were also found to have a better antitumor profile than the standard drugs harmine and cisplatin. 

Similarly, in “thiazole as antimicrobial agents,” many thiazole ring-containing compounds showed higher antimicrobial activity than the standard drugs used. For example, in one study, synthesized molecules **39a** and **39b** demonstrated a better activity profile when compared to the reference drugs ampicillin and streptomycin. In another study, one novel thiazole compound **40** was found equipotent to chloramphenicol against S. aureus (MIC 3.125 g/mL) and considerable activity against B. thuringiensis (MIC 6.25 micro-g/mL). In a separate study, the derivative 5-(4H-imidazo [1,2-b][1,2,4]triazol-5-yl)-N,4-dimethylthiazol-2-amine **42** exceeded the activity profile of the standard (amphotericin B) against Staphylococcus epidermidis. In one important study, novel thiazole-based chalcones **50** appeared to be 10- and 56-fold more potent compared to streptomycin and ampicillin, respectively. Another study reported the compound 3-(4-(adamantan-1-yl)thiazol-2-yl)-2-(2,3-dichlorophenyl)thiazolidin-4-one **52** to havee MICs of 0.9–6.25 µmol mL^−1^ × 10^−2^ and MBCs of 1.53–12.5 µmol mL^−1^ × 10^−2^. Compound **52** also displayed high fungistatic activity against all of the investigated fungi, with MIC values as low as 0.021–0.042 µmol mL^−1^ × 10^−2^ and MFC values as low as 0.06 µmol mL^−1^ × 10^−2^.

Thiazoles as antitubercular agents have received wider attention. Many new derivatives were synthesized to combat multidrug-resistant Mycobacterium tuberculosis. A majority of the compounds reported in this review displayed moderate to good activity in combating the resistant species. In one study, substituted 4-arylthiazol-2-amino derivatives as modified analogs of nitazoxanide (NTZ) **70a** (MIC = 15.28 µM) and **70b** (MIC = 17.03 µM) showed almost three times stronger Mtb growth inhibitory action than NTZ and were free of cytotoxicity (Vero CC50 of 244 and 300 µM, respectively).

In pursuit of finding new anti-inflammatory agents, several thiazole derivatives were synthesized during the last decade. Many of them were moderate to good in controlling inflammation; however, some displayed better results than the standard drugs. In one report, some new substituted pyrazoles containing thiazolyl and thiazolidinonyl derivatives **73a**, **73b, 73c**, **73d**, **73e**, and **73f** exhibited better anti-inflammatory efficacy than the reference drug celecoxib. In another study, a different class of tetrahydronaphthalene–thiazole-coumarin multi-nucleus derivative (E)-3-((2-(5-(5,6,7,8-tetrahydronaphthalen-2-yl)thiazol-2-yl)hydrazono)methyl)-4H-chromen-4-one **76** displayed higher anti-inflammatory and analgesic activity than indomethacin.

In the subsection “thiazoles as antimalarial agents,” pyrazole-linked thiazole analogs were found to be promising against Plasmodium berghei and chloroquine-inactive (RKL9) Plasmodium falciparum strains. The results highlighted the greatest potency was achieved by molecule **89** along with a few other compounds.

Though many thiazole-bearing antiviral derivatives were synthesized in the recent past, the activities of the majority of the derivatives were not found to be superior to standard antiviral drugs. In a report, compounds 101a, **101c**, and **101d** showed superior antiviral activity against EV71, and compounds **101a**, **101b**, **101c**, **101d**, **102a**, and **102b** had superior antiviral activity against CVB3, when compared to the reference drugs ribavirin or pirodavir.

In the subsection thiazoles as anti-Alzheimer agents, some newly synthesized derivatives were moderately good at eliciting anti-Alzheimer’s activity. The most active molecules against AChE were reported to be **111**, with an IC_50_ of 0.011 µM, whereas the IC_50_ value of the conventional medication donepezil was 0.054 µM. In the other study, compound 123 was found to be the most active agent in the series with an IC_50_ value of 0.028 ± 0.001 µM, which indicated an inhibition profile similar to the reference drug, donepezil.

In the subsection of thiazoles as anti-diabetic agents, the majority of the newly synthesized compounds exhibited good activity. After a critical analysis, **134** were observed to be a more potent inhibitor of DPP-4 (2.32 nM) than the standard alogliptin (3.56 nM).

Many thiazole-bearing compounds displayed moderately good adenosine receptor inhibitory activity. However, compound **141** showed very strong A2AR selectivity in comparison to the conventional A2AR antagonist SCH58261. In another study, compound **142** was recommended as a lead structure for the development of second-generation non-xanthine-derived A1antagonists with innovative therapeutic potential.

In the last section, we reported some bioactive compounds with varied biological activities. Some compounds were found to be better than the respective reference drugs. For example, the selenourea derivatives of thiazole **145a**, **145b**, **145c**, and **145d** were proven to have better antioxidant activity (AO) and lower IC_50_ values than the reference drugs. In a separate study, disubstituted thiazole derivative **147** displayed higher antioxidant properties than the standard BHT. In another study on insect repellent activity, analogous **152a**, **152b**, and **152c** exhibited the highest repellent activity comparable to the positive control DEET. In a separate study, a thiazole derivative called 4,5-bis(p-methoxyphenyl)-2-(trifluoromethyl)-thiazole **153** was found to be a potent suppressor of collagen-induced platelet aggregation. At a concentration of 1 ng/mL, it was effective in human platelet-rich plasma, which was superior to the standard drug flurbiprofen.

The review at a glance indicated that the thiazole moiety alone displayed a host of diverse pharmacological activities. All these thiazole-bearing derivatives needed further investigation and optimization. The toxicity profile should also be investigated as, in many reports, a toxicity study was missing. However, further investigations of some of the highly potent derivatives may lead to the generation of drug candidates.

## Figures and Tables

**Table 1:** Thiazole-ring-bearing drug candidates under intensive preclinical/clinical investigations.

Bioactive Compounds	Remarks/Conclusions
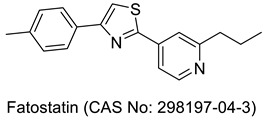	Fatostatin (125B11): A specific inhibitor of sterol response element-binding proteins (SREBPs) is a newly customized thiazole analogue combined with two aryl groups. Fatostatin inhibits proliferation and increases cellular deaths in cancer cells. The drug may possibly be prescribed in the therapy of uterine carcinoma [44].
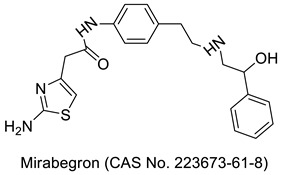	Mirabegron (YM 178): The medication is a selective β3-adrenoceptor stimulant that was recently licensed to alleviate overactive bladder (OAB) [45].
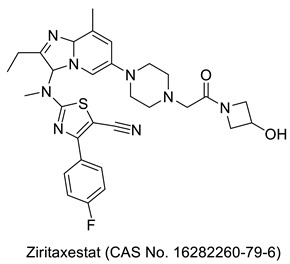	Ziritaxestat (GLPG1690): An autotaxin inhibitor is being optimized as a potential therapeutic candidate. The medication is still in development because phase 2 clinical studies for the clinical use of Systemic Sclerosis were unsuccessful [46,47].
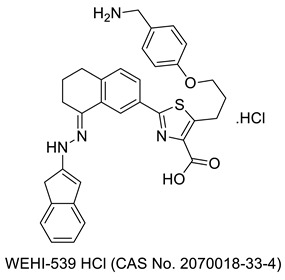	WEHI-539 HCl (WEHI-539 hydrochloride): The therapeutic candidate binds to BCL-XL with great affinity and specificity, killing cells effectively by inhibiting its pro-survival function. The molecule is in the developmental stage and is currently being optimized to achieve better physicochemical parameters. If further developed, the medicine could be approved for the management of a host of drug-resistant tumors [48].
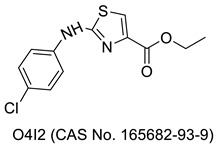	O4I2: The drug candidate is a powerful Oct3/4 inducer that possibly be prescribed to make iPSCs. This molecule is in a preclinical developmental stage and may possibly be used in the treatment of a variety of cancer [49].
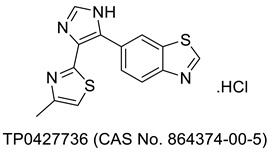	TP0427736: The compound is an effective inhibitor of ALK5 kinase activity. It also suppresses TGF-β1-induced phosphorylation of Smad2/3 in A549 cells. The drug is being optimized for its potential use in androgenic alopecia [50].
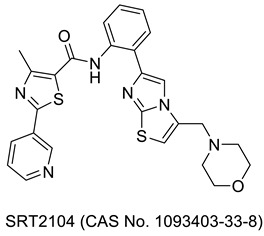	SRT2104 (GSK2245840): The compound is a selective Sirtuin 1(SIRT1) activator engaged in energy homeostasis management. The drug candidate is being investigated for clinical use for Atrophy, Sepsis, Psoriasis, Type 2 Diabetes Mellitus, and Muscular Dystrophy [51,52].
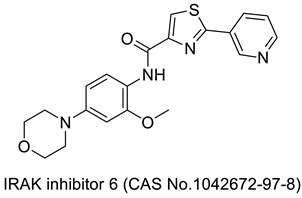	IRAK inhibitor 6: The therapeutic candidate is a selective Renal Carcinoma Antigen NY REN 64 blocker that can possibly be used to manage bone degradation and rheumatoid arthritis-induced joint inflammation [53].
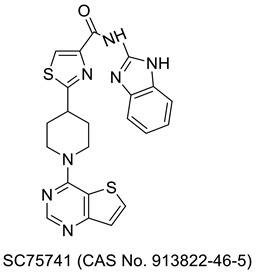	SC75741: The therapeutic candidate is a highly effective NF-B inhibitor. Due to its efficacy at inhibiting influenza virus replication, SC75741 is being developed to treat avian influenza-A virus infections [54].
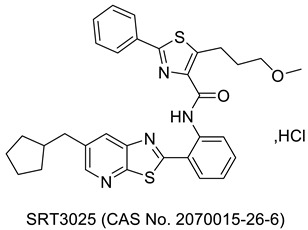	SRT3025: The active agent is a small molecule activator of the SIRT1 enzyme. The therapy could be used to manage Fanconi anaemia [55].
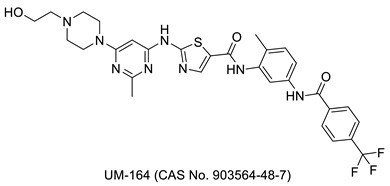	UM-164: The active substance is a highly powerful dual c-Src/p38 inhibitor that inhibits both p38 and p38 and has a binding constant Kd of 2.7 nM for c-Src. The drug is the subject of a great deal of research since it claims to be able to cure triple-negative breast cancer [56].
